# Chen’s Ricci inequalities and topological obstructions on null hypersurfaces of a Lorentzian manifold

**DOI:** 10.1186/s13660-018-1714-y

**Published:** 2018-05-29

**Authors:** Karimumuryango Ménédore

**Affiliations:** Institut de Mathématiques et de Sciences Physiques (IMSP), Porto-Novo, Bénin

**Keywords:** Null hypersurface, Rigging, Closed normalization, Associated Riemannian metric, Ricci inequalities, Minimal hypersurface

## Abstract

Given a null hypersurface of a Lorentzian manifold, we isometrically immerse a null hypersurface equipped with the Riemannian metric (induced on it by the rigging) into a Riemannian manifold suitably constructed on the Lorentzian manifold. We study the intrinsic and extrinsic geometry of such an isometric immersion and we link them to the null geometry of the null hypersurface in the Lorentzian manifold. In the course of this immersion, we find the basic relationships between the main extrinsic invariants and the main intrinsic invariants, named Chen-Ricci inequalities of the null hypersurface in the Lorentzian manifold. The findings prove a topological implication of these relationships.

## Introduction

Soon after Riemann (1854) introduced the notion of a manifold, Schläfli (1873) conjectured that every Riemannian manifold could be locally considered as a submanifold of an Euclidean space with sufficiently high codimension. This was later proved in different steps by Janet (1926), Cartan (1927), Burstin (1931) and extended to semi-Riemannian manifolds by Friedmann (1965). In 1956, Nash proved that every *n*-Riemannian manifold can be isometrically embedded in an Euclidean *m*-space $\Bbb{E}^{m}$ with $m=\frac{n}{2}(n+1)(3n+11)$. From the aforementioned, it is difficult to apply Nash’ theorem, because it requires a very large codimension and there is no general optimal relationships between the known intrinsic invariants and the main extrinsic invariants for Riemannian submanifold of Euclidean spaces. To overcome the difficulties Chen introduced in 1993 a new type of Riemannian invariants for a Riemannian manifold *M* [6]. We have

1 $$ \delta_{M}=\tau(p)-\inf K(p), $$

where $\tau(p)$ is scalar curvature of *M* and $\inf(K)(p) = \inf\{K(\pi): K(\pi)$ is a plane section of $T_{p}M \}$.

In [5], Chen established the following general optimal inequality involving the new intrinsic invariant $\delta_{M} $ and the squared mean curvature $\| H\|^{2}$ for *n*-dimensional submanifold *M* of a real space form of constant sectional curvature *c*:

2 $$ \delta_{M}\leq\frac{n^{2}(n-2)}{2(n-1)} \Vert H \Vert ^{2}+ \frac{1}{2}(n+1) (n-2)c. $$

In [8], Chen proved a basic inequality involving the Ricci curvature and the squared mean curvature of a submanifold of a real space form:

3 $$ \operatorname{Ric}(X)\leq\frac{1}{4}n^{2} \Vert H \Vert ^{2}+(n-1)c. $$

In [7], B.Y. Chen estabished the inequality between the shape operator and the mean curvature of isometric immersions in real space forms. In [16], the autors give some remarks on B.Y. Chen’s inequality involving classical invariants. In [13] Hong and Tripathi studied this inequality and they presented a general theory for submanifolds of Riemannian manifolds and proved a basic inequality using (3) as follows:

4 $$ \operatorname{Ric}(X)\leq\frac{1}{4}n^{2} \Vert H \Vert ^{2}+\overline {\operatorname {Ric}}_{(T_{p}M)}(X), $$

where *M* is a *n*-dimensional submanifold of *M̅*, $\operatorname{Ric}_{(T_{p}M )} (X)$ is the *n*-Ricci curvature of $T_{p} M$ at $X\in T^{1}_{p} M$ with respect to the ambient manifold *M̅* and $T^{1}_{p} M$ is the set of unit vectors in $T_{p}M$. The equality case of (4) is satisfied by $X \in T^{1}_{p} M$ if and only if

5 $$ \left \{ \textstyle\begin{array}{l} \alpha(X,Y)=0,\quad\forall Y\in T_{p}M, \qquad g(X,Y)= 0,\\ 2\alpha(X,X)= n H. \end{array}\displaystyle \right . $$

**Figure 1:**
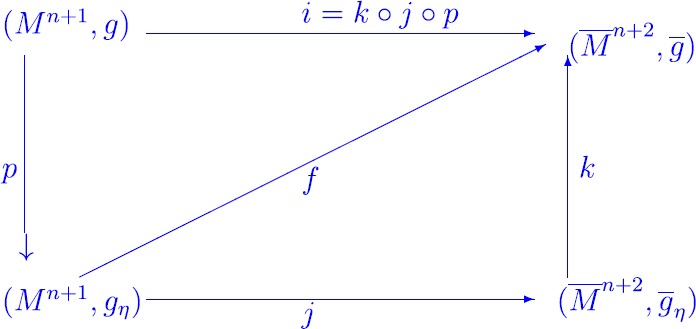
Links between the null and Lorentzian geometry

In [18], this inequality was named a Chen–Ricci inequality by Tripathi where *α* is the second fundamental form of *M*. The equality case of (4) holds for all unit vectors $X \in T^{1}_{p} M $ and for all $p \in M $ if and only if either *M* is totally geodesic, or $n=2$ and *p* is a totally umbilical point. In the degenerate submanifolds, Gülbahar, Kiliç and Keleş introduced k-Ricci curvature, k-scalar curvature, k-degenerate Ricci curvature, k-degenerate scalar curvature and they established some inequalities that characterize lightlike hypersurfaces of a Lorentzian manifold ([10]). Afterward, in [11] they established some inequalities involving k-Ricci curvature, k-scalar curvature, the screen scalar curvature on a screen homothetic lightlike hypersurface of a Lorentzian manifold and they computed the Chen–Ricci inequality and the Chen inequality on a screen homothetic lightlike hypersurface of a Lorentzian manifold. In [15], they established some inequalities on the sectional curvature of lightlike submanifolds. In this present paper, we first consider the associated Riemannian metric of a null hypersurface in a Lorentzian manifold as in [3] but arising from a null rigging defined on a neighborhood of the null hypersurface, and we isometrically immerse the null hypersurface equipped with the associated Riemannian metric (induced on it by the rigging) into a Riemannian manifold suitably constructed on the Lorentzian manifold. We establish the link between intrinsic and extrinsic geometry of such isometric immersion (Proposition 4.1, Theorem 4.1). We connect the geometry of the Riemannian metric constructed on the Lorentzian ambient manifold with the Lorentzian geometry (Theorem 4.2, Theorem 4.3, Theorem 4.4, Theorem 4.5). We establish the Chen inequalities of a null hypersurface $M^{n+1}$ in a Lorentzian ambient manifold $\overline{M}^{n+2}$ (Theorem 5.1, Theorem 5.2, Theorem 5.3). It is here important first to give the organizational structure of this paper. Section 2 gives the necessary preliminaries about null hypersurfaces. In Sect. 3, we give the links between a null geometry and its associated Riemannian geometry. The relationships between the intrinsic and extrinsic geometries of the immersion of the null hypersurface $M^{n+1}$ equipped with the associated Riemannian metric (induced on it by the rigging) into a Riemannian manifold suitably constructed on the Lorentzian ambient manifold $\overline{M}^{n+2}$ and the relations between the Riemannian geometry constructed on the Lorentzian manifold and the Lorentzian geometry are discussed in Sect. 4. We connect the intrinsic and extrinsic geometries of this immersion in order to link those two geometries to the null geometry and the Lorentzian one (see Fig. 1).

In the last section, we establish Chen’s inequalities for a null hypersurface in Lorentzian manifold and we discover a topological obstruction to the minimal isometric immersion of the null hypersurface in an ambient Lorentzian manifold.

## Preliminaries

Consider a null hypersurface $(M^{n+1},g)$ of a $(n+2)$-dimensional Lorentzian manifold $(\overline{M},\overline{g})$ of constant index $0<\nu< n+2$. The normal bundle of the null hypersurface is the subbundle $TM^{\bot}= \{ V\in\Gamma(T\overline{M}): g(V,W)= 0\ \forall W\in\Gamma(T{M})\} $ of the tangent bundle *TM*. Since *M* is a null hypersurface, $\dim(T_{x}M^{\bot})=1$.

In the classical theory of non-degenerate hypersurfaces, we have the following decomposition:

6 $$ T\overline{M}=TM \oplus_{\mathrm{orth}}TM^{\bot},\qquad TM\cap TM^{\bot}=\{0\}, $$

where $\oplus_{\mathrm{orth}}$ denotes orthogonal direct sum. Any vector field of *TM̅* splits uniquely into a component tangent to *M* and a component perpendicular to *M*. However, in the null hypersurface case, (6) does not hold because *TM* and $TM^{\bot}$ have a non-trivial intersection. Therefore, the introduction of the main induced geometric objects on *M* as the Levi-Civita connection, the second fundamental form, the operator form, have different properties from the non-degenerate case. In [9], the authors introduced a complementary bundle of $TM^{\bot}$ in *TM* which is a rank *n* non-degenerate distribution over *M*, called a screen distribution of *M*, which we denote by $\mathscr{S}(N)$, such as

7 $$ TM = \mathscr{S}(N) \oplus_{\mathrm{orth}} TM^{\bot}. $$

The existence of $\mathscr{S}(N) $ is secured provided that *M* is paracompact. A null hypersurface with a specific screen distribution is given by $( M,g,\mathscr{S}(N))$. It is well known from [9] that, for such a triplet $(M,g,\mathscr{S}(N))$, there exists a unique rank 1 vector subbundle $\operatorname{tr}(T M )$ of *TM̅* over *M*, such that, for any non-zero section *ξ* of $T M^{\bot}$ on a coordinate neighborhood $\mathscr{U} \subset M$, there exists a unique section *N* of $\operatorname{tr}(T M )$ on $\mathscr{U}$ satisfying

8 $$ \bar{g}(N,\xi)=1, \qquad\bar{g}(N,N)=\bar{g}(N,W)= 0,\quad \forall W\in\Gamma( \mathscr{S}N)_{|_{\mathscr{U}}}. $$

Then *TM̅* is decomposed as follows:

9 $$ T \bar{M}= \mathscr{S}(N)\oplus_{\mathrm{orth}} \bigl(TM^{\bot}\oplus \operatorname {tr}(TM) \bigr) = TM \oplus \operatorname{tr}(TM), $$

$\operatorname{tr}(TM)$ is called a (null) transversal vector bundle along *M*. In fact, from (8) and (9) one shows that, conversely, a choice of a transversal bundle $\operatorname{tr}(TM)$ determines uniquely the distribution $\mathscr{S}(N)$. A vector field *N* as defined in (8) is called a null rigging of *M*. It is noteworthy that the choice of a null transversal vector field *N* along *M* determines the null transversal vector bundle, the screen distribution and a unique radical vector field *ξ*, say the rigged vector field, satisfying (8).

### Definition 2.1

([12])

Let *M* be a null hypersurface of a Lorentzian manifold. A rigging for *M* is a vector field *L* defined on some open set containing *M* such that $L_{p} \notin T_{p} M$ for each $p \in M$.

An outstanding property of a rigging is that it allows a definition of geometric objects globally on *M*. We say that we have a null rigging when the restriction of *L* to the null hypersurface is a null vector field. Throughout the paper, we fix a null rigging *N* for *M* on *M̅*. In particular this rigging fixes a unique null vector field $\xi\in\Gamma(TM^{\bot})$ called the rigged vector field. From now on, we denote the normalized (or rigged) null hypersurface by a triplet $(M,g,N )$ where $g = \overline{g}_{|M}$ is the first fundamental form and *N* is a null rigging for *M*. Let *N* be a null rigging of a null hypersurface of a Lorentzian manifold $(\overline{M}^{n+2},\overline{g})$ and *θ* the 1-form metrically equivalent to *N* defined on some open set containing *M* and given by

10 $$ \theta=\overline{g}(N,\cdot). $$

Suppose that

11 $$ \eta= i^{\star}{\theta} $$

is a restriction to *M*, $i: M\rightarrow\overline{M} $ being the inclusion map. The normalization *N* will be said to be closed if the 1-form *θ* is closed on *M*. It is easy to check that $\mathscr{S}(N) = \operatorname{ker}(\eta)$ and the screen distribution $\mathscr{S}(N)$ is integrable whenever *η* is closed. On a normalized null hypersurface $(M, g, N )$, the Gauss and Weingarten type formulae are given by, respectively,

12 $$\begin{aligned}& \overline{\nabla}_{X}Y=\nabla_{X}Y + B^{N}(X,Y)N, \end{aligned}$$

13 $$\begin{aligned}& \overline{ \nabla}_{X}N=- A_{N}X + \tau^{N}(X)N, \end{aligned}$$

14 $$\begin{aligned}& \nabla_{X}PY=\nabla^{*}_{X}PY +C^{N}(X,PY)\xi, \end{aligned}$$

15 $$\begin{aligned}& \nabla_{X}\xi=-A^{*}_{\xi}X - \tau^{N}(X)\xi \end{aligned}$$

for any $X,Y\in\Gamma(TM)$, where ∇̅ denotes the Levi-Civita connection on $(\overline{M},\overline{g})$, ∇ denotes the rigged connection on $(M,g)$ induced from ∇̅ through the projection along *N*; it satisfies

16 $$ (\nabla_{X} g) (Y,Z) = B^{N}(X,Y)\eta(Z) +B^{N}(X,Z)\eta(Y), $$

and $\nabla^{*}$ denotes the induced connection on the screen distribution.

Here *η* is an 1-form on *TM* defined by

17 $$ \eta(X) = \overline{g} (N,X),\quad\forall X \in\Gamma(TM), $$

$B^{N} $ is the null second fundamental form of *M* and $C^{N} $ is the second fundamental on $(\mathscr{S}(N))$, respectively. The second fundamental forms are related by their shape operators,

18 $$\begin{aligned}& B^{N}(X,Y)=g\bigl(A^{*}_{\xi}X,Y\bigr),\qquad \overline{g}\bigl(A^{*}_{\xi}X,N\bigr)=0, \end{aligned}$$

19 $$\begin{aligned}& C^{N}(X,PY)=g(A_{N}X,PY), \qquad\overline{g}(A_{N}Y,N)=0,\quad \forall X,Y\in\Gamma(TM). \end{aligned}$$

$A^{*}_{\xi}$ is the null shape operator with respect to the section *ξ* and

20 $$ B^{N}(X,\xi)= 0 ,\qquad A^{\star}_{\xi}\xi=0 . $$

$\tau^{N} $ is the 1-form on *TM* defined by $\tau^{N}(X)=\overline{g}(\overline{\nabla}_{X}N,\xi)$. From (15) and $A^{*}_{\xi}\xi=0$, we find that

21 $$ \nabla_{\xi}\xi=-\tau^{N}(\xi)\xi, $$

which means that integral curves of *ξ* are pregeodesic. Throughout the paper, we consider the integral curves of *ξ* to be geodesics in *M̅* and *M*, which means that

22 $$ \tau^{N}(\xi)=0. $$

### Example 2.1

Let $\mathscr{F} $ be the immersion $\mathscr{f}:M^{n+1}_{0 } \rightarrow\Bbb{R}_{1}^{n+2}$ defined by $\mathscr{F}(x^{1},\ldots,x^{n})\mapsto[x^{1},\ldots,x^{n},(x^{1})^{2} + \cdots+(x^{n})^{2}]$, and the null hypersurface $M_{0} ^{n+1}= \{x = (x_{0},\ldots,x_{n+1}), -x_{0}^{2} + \sum^{n+1}_{a=1} x_{a}^{2} = 0\}$. Let *N* be the null rigging of *M* defined by $N = -x_{0}\partial_{0} + \sum^{n+1}_{a=1}x_{a}\partial_{a}$; and the null vector field $\xi= \frac{1}{2x^{2}_{0}} (x_{0}\partial_{0} + \sum^{n+1}_{a=1}x_{a}\partial_{a} )$, $x_{0}\neq0$. Hence the null vector field *ξ* is normal to *M*, thus *M* is a null hypersurface.

Let $X = X_{0}\partial_{0} + \sum^{n+1}_{b=1} X_{b}\partial_{b}$, the 1-form *η* is given by

$$\begin{aligned} \eta(X)&=\langle N,X\rangle \\ &= \Biggl\langle -x_{0}\partial_{0} + \sum ^{n+1}_{a=1}x_{a}\partial _{a},X_{0}\partial_{0} + \sum ^{n+1}_{b=1}X_{b}\partial_{b} \Biggr\rangle \\ &=\Biggl(x_{0}X_{0} + \sum^{n+1}_{a=1}x_{a}X_{a} \Biggr) \\ &=2x_{0}\,dx_{0}(X).\end{aligned} $$

Hence $\eta= 2x_{0}\,dx_{0}$ and $d\eta= 0 $. This shows that *η* is closed and $\tau^{N}(X) = - [2x^{2}_{0} (-\frac{dx_{0}}{x^{3}_{0}})+\frac{1}{2x^{2}_{0}} 2x_{0}\, dx_{0} ](X)$ for all $X \in\Gamma(T M)$.

### Definition 2.2

A null hypersurface *M* is said to be totally umbilical (respectively, totally geodesic) if there exists a smooth function *ρ* on *M* such that at each $x \in M $ and for all $X, Y \in T_{x} M$, $B^{N} (x)(X, Y) = \rho(x)g(X, Y)$ (respectively, $B^{N}$ vanishes identically on *M*). This is equivalent to write $A_{\xi}= \rho P$ and $A_{\xi}= 0$. Also, the screen distribution $\mathscr{S}(N)$ is totally umbilical (respectively, totally geodesic) if $C^{N} (X, P Y ) = \rho(x) g(X, Y )$ for all $X, Y \in\Gamma(T M )$ (respectively, $C^{N} = 0$), which is equivalent to writing $A_{N} = \rho P$ (respectively, $A_{N} = 0$).

Denote by *R̅* and *R* the Riemann curvature tensors of ∇̅ and ∇, respectively. Recall the following Gauss–Codazzi equations ([9]) for all $X,Y,Z \in\Gamma(TM)$, $N\in\operatorname{tr}(TM)$, $\xi\in\Gamma(TM^{\bot})$:

23 $$\begin{aligned}& \begin{aligned}[b]\overline{g}\bigl(\overline{R}(X,Y)Z,\xi\bigr) ={}&\bigl( \nabla_{X}B^{N}\bigr) (Y,Z) -\bigl( \nabla_{Y}B^{N} \bigr) (X,Z) +B^{N}(Y,Z)\tau^{N} (X) \\ &-B^{N}(X,Z)\tau^{N}(Y);\end{aligned} \end{aligned}$$

24 $$\begin{aligned}& \begin{aligned}[b]\bar{g}\bigl(\bar{R}(X,Y)\xi,N\bigr) &= \overline{g}\bigl(R(X,Y)\xi,N\bigr) \\ &=C^{N}\bigl(Y,A^{*}_{\xi}X\bigr)-C^{N}\bigl( X,A^{*}_{\xi}Y\bigr)-2 \,d\tau^{N}(X,Y);\end{aligned} \end{aligned}$$

25 $$\begin{aligned}& \begin{aligned}[b]\overline{g}\bigl(\overline{R}(X,Y)PZ,N\bigr) ={}&\bigl( \nabla_{X}C^{N}\bigr) (Y,PZ)-\bigl( \nabla_{Y}C^{N} \bigr) (X,PZ) \\ &+\tau^{N}(Y) C^{N}(X,PZ)-\tau^{N} (X)C^{N}(Y,PZ).\end{aligned} \end{aligned}$$

The shape operator $A_{\xi}^{\star}$ is self-adjoint as the second fundamental form $B^{N}$is symmetric. However, this is not the case for the operator $A_{N}$ as show in the following lemma.

### Lemma 2.1

*For all* $X,Y\in\Gamma(TM)$

26 $$ \langle A_{N} X ,Y \rangle-\langle A_{N}Y,X \rangle=\tau^{N}(X)\eta (Y)-\tau ^{N}(Y)\eta(X)-2\,d\eta(X,Y), $$

*where* (*throughout*) $\langle,\rangle=\overline{g}$ *stands for the Lorentzian metric*.

### Proof

Recall that $\eta= i^{\star}\theta$ where $\theta=\langle N,\cdot\rangle$. Taking the differential of *θ* and using the Weingarten formula, we have, for all $X,Y\in\Gamma(TM)$,

$$\begin{aligned} 2\,d\eta(X,Y)&=2\,d\theta(X,Y) \\ &=\langle\overline{\nabla}_{X}N,Y\rangle- \langle\overline{\nabla}_{Y}N,X\rangle \\ &=-\langle A_{N}X,Y\rangle+\tau^{N}(X)\eta(Y)+\langle A_{N} Y,YX\rangle -\tau(Y)\eta(X).\end{aligned} $$

Hence

27 $$ \langle A_{N}X,Y\rangle-\langle A_{N} Y,X\rangle = \tau^{N}(X)\eta(Y)-\tau^{N}(Y)\eta(X) $$

as announced. □

We shall use the following notations:

### Notations 2.1

For $x \in M $, we set $\mathscr{S}^{0}_{x}(1)=\{X\in T_{x}M, \langle X,X\rangle=1-\eta(X)^{2}\}$, $\mathscr{S}^{0}(1)= \bigcup_{x\in M}\mathscr{S}^{0}_{x}(1)$, and for all $X \in\Gamma(TM)$, we set $O_{\eta}(X)= \{Y\in TM, \langle X,Y\rangle=-\eta(X)\eta (Y) \}$, where $\langle,\rangle$ stands for *g̅* or *g*. Observe that $Y\in O_{\eta}(X)$ if and only if $X\in O_{\eta}(Y)$.

Due to the degeneracy of the first fundamental form *g* on the null hypersurface *M*, it is not possible to define the natural dual isomorphism between the tangent vector bundle *TM* and the cotangent vector bundle $T^{\star}M $ following the usual Riemannian way. However, this construction is possible by setting a rigging *N* (see [3], [1] for further details). Consider a normalized null hypersurface $(M,g,N) $ and 1-form defined by (17). For all $X\in\Gamma(TM)$, $X =PX +\eta(X)\xi$ and $\eta(X)=0 $ if and ony if $X\in\Gamma(\mathscr{S}(N))$. Now, we define $\flat_{\eta}$ by

28 $$ \begin{aligned} \flat_{\eta}:\Gamma(TM) & \rightarrow\Gamma\bigl(TM^{*}\bigr), \\ X &\mapsto X^{\flat_{\eta}} = g(X,\cdot) + \eta(X)\eta(\cdot),\quad \forall X \in \Gamma(T M), \\ X^{\flat_{\eta}}(Y)&= g(X,Y) +\eta(X)\eta(Y). \end{aligned} $$

$\flat_{\eta}$ is an isomorphism of $\Gamma(TM)$ on to $\Gamma(T^{*}M)$ and can be used to generalize the usual non-degenerate theory. In the latter case, $\Gamma(\mathscr{S}(N))$ coincides with $\Gamma (TM)$, and as a consequence the 1-form *η* vanishes identically and the projection morphism *P* becomes the identity map on $\Gamma(TM)$. Let $\sharp_{\eta}$ denote the inverse of the isomorphism $\flat_{\eta}$ given by (28). For $X\in\Gamma(TM)$ (respectively, $w \in T^{*}M$), $X^{\flat_{\eta}}$ (respectively, $w^{\sharp_{\eta}}$) is called the dual 1-form of *X* (respectively, the dual vector field of *w*) with respect to the degenerate metric *g*. It follows from (28) that if *w* is a 1-form on *M*, we have, for $X\in\Gamma(TM)$,

29 $$ w(X)=g\bigl(w^{\sharp_{\eta}},X\bigr)+ w(\xi)\eta(X). $$

Define a $(0,2)$-tensor $g_{\eta}$ by $g_{\eta}(X,Y)= X^{\flat_{\eta}} (Y)$, $\forall X,Y \in\Gamma(TM)$. Clearly, $g_{\eta}$ defines a non-degenerate metric on *M* which plays an important role in defining the usual differential operators (gradient, divergence, Laplacian) with respect to degenerate metric *g* on null hypersurface (for details see [3]). In this case, $g_{\eta}$ is called the associated metric to *g* on $(M,g,N)$. Also, observe that $g_{\eta}$ coincides with *g* if the latter is non-degenerate. The $(0,2)$-tensor $g_{\eta}^{-1}$, the inverse of $g_{\eta}$, is called the pseudo-inverse of *g* with respect to the rigging *N*.

With the quasi-orthonormal local frame field $\{\partial_{0}:=\xi, \partial_{1} , \ldots, \partial_{n} , N \}$ adapted to the decomposition (6) and (7) we have

30 $$\begin{aligned}[b] &g_{\eta}(X,Y)= g( X,Y ),\quad \forall X,Y\in\Gamma\bigl( \mathscr {S}(N) \bigr). \\ & g_{\eta}(\xi,X)= \eta(X) , \quad\forall X\in\Gamma( TM). \\ & g_{\eta}(\xi,\xi)=1.\end{aligned} $$

### Definition 2.3

([2])

A normalized null hypersurface $(M,g,N)$ of a pseudo-Riemannian manifold $(\overline{M}, \overline{g})$ is said to have a conformal screen if there exists a non-vanishing smooth function *φ* on *M* such that $A_{N}=\varphi A_{\xi}^{\star}$ holds.

This is equivalent to the fact that $C^{N}(X,PY)=\varphi B^{N}(X,Y)$ for all tangent vector fields *X* and *Y*. The function *φ* is called the conformal factor.

### Remark 2.1

For all $x\in M $

31 $$ \mathscr{S}^{0}_{x} (1) = \bigl\{ X \in T_{x} M, \langle X, X \rangle= 1-\eta (X)^{2} \bigr\} = \bigl\{ X\in T_{x}M, g_{\eta}(X,X) = 1\bigr\} , $$

that is, $\mathscr{S}^{0} (1)$ coincides with the unit bundle of *M* with respect to the associated Riemannian metric $g_{\eta}$ from the normalization. Also, for all $X \in T_{x} M$, $O_{\eta}(X) = X^{{\bot}_{g_{\eta}}}$.

## Relations between null and the associated Riemannian geometry

Let $(M,g,N)$ be a normalized null hypersurface of a pseudo-Riemannian manifold $(\overline{M}^{n+2},\overline{g})$, ∇ the induced connection on *M*. In order to relate the main geometric objects of both null and associated non-degenerate geometry on the null hypersurface, we first need to relate the covariant derivatives $\nabla^{\eta}$ and ∇. For this purpose, we recall the following.

### Lemma 3.1

([3])

*For all* $X,Y,Z\in\Gamma(TM)$, *we have*

32 $$ \begin{aligned}[b](\nabla_{X} g_{\eta}) (Y,Z) ={}&\eta(Y) \bigl[B^{N}(X,PZ)-C^{N}(X,PZ)\bigr] \\ &+ \eta(Z)\bigl[B^{N}(X,PY)-C^{N}(X,PY)\bigr] \\ &+2\tau^{N}(X)\eta(Y)\eta(Z).\end{aligned} $$

In this respect and using Lemma 3.1 we give the following result.

### Proposition 3.1

([14])

*Let* $(M,g,N)$ *be a normalized null hypersurface with rigged vector field* *ξ*. *Then*, *for all* $X,Y \in\Gamma(TM)$, *we have*

33 $$ \begin{aligned}[b]\nabla^{\eta}_{X}Y={}&\nabla_{X}Y + \frac{1}{2} \bigl[ 2 g\bigl(A^{*}_{\xi}X,Y\bigr)-g(A_{N}X,Y)-g(A_{N}Y,X) \\ &+\eta(X)\tau^{N}(Y)+\eta(Y)\tau^{N}(X) \bigr]\xi + \eta(X) (i_{Y}\,d\eta)^{\sharp_{\eta}}+\eta(Y) (i_{X}\,d \eta)^{\sharp _{\eta}}.\end{aligned} $$

*In particular for a closed normalization*

34 $$ \begin{aligned}[b]\nabla^{\eta}_{X}Y={}&\nabla_{X}Y + \frac{1}{2} \bigl[ 2 g \bigl(A^{*}_{\xi}X,Y\bigr) - g(A_{N}X,Y)-g (A_{N}Y,X) \\ & +\eta(X)\tau^{N}(Y)+\eta(Y)\tau^{N}(X) \bigr]\xi\end{aligned} $$

*with* $d\eta(X,Y) = \frac{1}{2} [X\cdot \eta(Y) - Y\cdot\eta(X)-\eta([X,Y]) ]$.

Using Proposition 3.1 we prove the following.

### Proposition 3.2

*Let* $(M, g, N )$ *be a closed normalized null hypersurface with rigged vector field* *ξ*. *Then*, *for all* $X, Y, W \in\Gamma(T M )$ *and* $U \in\Gamma(T M^{\bot})$, *we have*

35 $$\begin{aligned}& \begin{aligned}[b] g_{\eta}\bigl(R^{\eta}(X,Y)Z,PW\bigr) ={}& g\bigl( R(X,Y)Z,PW \bigr) \\ &+\frac{1}{2}\bigl\{ \phi_{(X,Z)}B^{N} (Y,W) - \phi_{(Y,Z)}B^{N}(X, W)\bigr\} ,\end{aligned} \end{aligned}$$

36 $$\begin{aligned}& \begin{aligned}[b]g_{\eta}\bigl(R^{\eta}(X,Y)Z,U\bigr) ={}&{-} g\bigl( R(X,Y)U,PZ \bigr) \\ &-\frac{1}{2} \bigl[ g(A_{N}U,Y)B^{N}( X,Z) - g(A_{N}U,X)B^{N}(Y,Z) \bigr] \\ &-\frac{1}{2} \bigl[\tau^{N}(X)B^{N}( Y,Z) - \tau^{N} (Y) B^{N}( X,Z) \bigr]\eta(U),\end{aligned} \end{aligned}$$

*where* $\phi(X,Z)$ *is given by*

37 $$\begin{aligned}[b] \phi_{(X,Z)}={}&2 B^{N}(X,Z) - g( A_{N}X,Z) -g(A_{N}Z,X) \\ &+\tau^{N}(X)\eta(Z) + \tau^{N}(Z)\eta(X).\end{aligned} $$

### Proof

The Riemann curvature tensor field $R^{\eta} $ of type $(1, 3)$ is defined by

38 $$ R^{\eta} (X, Y )Z =\bigl[\nabla^{\eta}_{X}, \nabla^{\eta}_{Y}\bigr]Z-\nabla ^{\eta}_{[X,Y]}Z. $$

Then (35) and (36) consist of repeated applications of (34) in Proposition 3.1. □

Let $\operatorname{Ric}^{\eta}$ and Ric denote the Ricci curvature of $( M,g_{\eta})$ and $(M,g,N)$, respectively. We shall give the following result, involving the extrinsic Ricci curvature Ric to the associated Ricci curvature $\operatorname{Ric}^{\eta}$.

### Theorem 3.1

*Let* $(M,g,N)$ *be a closed normalized null hypersurface with rigged vector field* *ξ* *and* $\tau^{N} (\xi)=0 $ *in a* $(n + 2)$-*pseudo*-*Riemannian manifold*. *Then*

39 $$ \begin{aligned}[b] \operatorname{Ric}^{\eta}(X,Y)={}& \operatorname{Ric}(X,Y) - \bigl[\bigl\langle A^{*}_{\xi}X,Y\bigr\rangle - \langle A_{N}X,Y\rangle+ \tau^{N}(X)\eta(Y) \bigr] \operatorname{tr}A^{*}_{\xi} \\ &+\bigl\langle \bigl(\nabla_{\xi}A^{*}_{\xi}\bigr) (X),Y \bigr\rangle -\bigl\langle (\nabla_{\xi}A_{N}) (X),Y \bigr\rangle \\ &+\bigl(\nabla_{\xi}\tau^{N}\bigr) (X)\eta(Y) - \bigl( \nabla_{X}\tau^{N}\bigr) (Y).\end{aligned} $$

### Proof

Let $p\in M$ and $(E_{0}:=\xi, E_{1},\ldots,E_{n})$ be a quasi-orthonormal basis for $(T_{p}M,g_{p})$ with $\operatorname{Span} (E_{1},\ldots,E_{n})=\mathscr {S}(N)_{p}$. When dealing with indices, we adopt the following conventions: $i,j,k,\ldots\in\{1,\ldots,n\}$, $\alpha,\beta,\gamma\in\{0,\ldots,n\}$, and $a,b,\ldots,\in\{ 0,\ldots ,n+1\}$. Then we have

40 $$ \operatorname{Ric}^{\eta}(X,Y)=\sum^{n}_{\alpha= 0}g^{\alpha\alpha }_{\eta}\big( R^{\eta}(E_{\alpha},X)Y,E_{\alpha}\big). $$

Thus, from (35) and (36), we get

$$\begin{aligned}& \begin{aligned} R^{\eta}ic(X,Y)={}&g_{\eta}\bigl(R^{\eta}(\xi,X)Y,\xi \bigr) + \sum^{n}_{i} g_{\eta} \bigl(R^{\eta}(E_{i},X)Y,E_{i}\bigr) \\ ={}& \operatorname{Ric}(X,Y)+ g\bigl(A^{*}_{\xi}X, A^{*}_{\xi}Y \bigr) - g\bigl(A^{*}_{\xi}X,Y\bigr) - g\bigl(A^{*}_{\xi}X,Y \bigr)\operatorname{tr}A^{*}_{\xi}\\ &- g\bigl(A^{*}_{\xi}X,A_{N}Y\bigr) + \frac{1}{2} \bigl[g(A_{N}X,Y) + g(A_{N}Y, X) - \tau^{N}(X) \eta(Y) \\ & - \tau^{N}(Y)\eta(X) \bigr]\operatorname{tr}A^{*}_{\xi}- g\bigl(R(\xi,X) \xi,Y\bigr) - \overline{g}\bigl(\overline{R}(\xi,X)Y,N\bigr),\end{aligned} \\& \begin{aligned}R^{\eta}ic(X,Y) ={}& \operatorname{Ric}(X,Y)- \bigl[ \bigl\langle A^{*}_{\xi}X,Y\bigr\rangle - \langle A_{N}X,Y\rangle \bigr]\operatorname{tr}A^{*}_{\xi}\\ &- \tau^{N}(X)\eta(Y)\operatorname{tr}A^{*}_{\xi}+ \bigl\langle X,\bigl( \nabla_{\xi}A^{*}_{\xi}\bigr) (Y)\bigr\rangle -\bigl( \nabla_{X}\tau^{N}\bigr) (Y) \\ & - \bigl\langle (\nabla_{\xi}A_{N}) (X),Y\bigr\rangle + 2d \tau^{N}(\xi,X)\eta(Y) + \tau^{N}\bigl(A^{*}_{\xi}X \bigr)\eta(Y),\end{aligned} \end{aligned}$$

where

41 $$ \operatorname{Ric}(X,Y)=\sum^{n}_{i=1} \varepsilon_{i} g\bigl(R(E_{i},X)Y,E_{i}\bigr) + \overline{g}\bigl(R(\xi,X)Y,N\bigr) $$

is the induced Ricci tensor curvature on a null hypersurface. But $\langle X,(\nabla_{\xi}A^{*}_{\xi})(Y)\rangle=\langle(\nabla_{\xi}A^{*}_{\xi})(X),Y\rangle$ and $2\,d\tau^{N}(\xi, X)=(\nabla_{\xi}\tau^{N})(X) -(\nabla_{X}\tau^{N} )(\xi ) $ and $(\nabla_{X}\tau^{N})(\xi)=\tau^{N}(A^{*}_{\xi}X) $. Also $g(R(\xi,X)\xi,Y)=g(A^{*}_{\xi}X,A^{*}_{\xi}Y)-g(X,(\nabla_{\xi}A^{*}_{\xi})(Y))$ and

$$ \begin{aligned}\overline{g}\bigl(\overline{R}(\xi,X)Y,N\bigr)={}& \bigl(\nabla_{X} \tau^{N}\bigr) (Y) + \bigl\langle (\nabla_{\xi}A_{N}) (X),Y\bigr\rangle \\ & -2\,d\tau^{N}(\xi,X)\eta(Y)-\bigl\langle A_{N}A^{*}_{\xi}X,Y\bigr\rangle .\end{aligned}$$

By substituting previous terms in the above expression of $\operatorname {Ric}^{\eta}(X,Y)$ we get the desired formula. □

For all $X,Y\in\Gamma(TM)$ we define the $(0, 2)$-symmetrized Ricci tensor Ric^0^ on the null hypersurface by

42 $$ \operatorname{Ric}^{0}(X,Y)=\frac{1}{2}\bigl\{ \operatorname {Ric}(X,Y)+\operatorname{Ric}(Y,X)\bigr\} . $$

### Theorem 3.2

*Let* $(M, g, N )$ *be a closed normalized null hypersurface with rigged vector field* *ξ* *and* $\tau^{N} (\xi) = 0 $ *in a pseudo*-*Riemannian manifold*. *Then*

43 $$ \begin{aligned}[b]r^{\eta}={}& r^{0} - \bigl[\operatorname{tr} A^{*}_{\xi}- \operatorname{tr} A_{N} \bigr]\operatorname{tr} A^{*}_{\xi}\\ &+ \bigl[ \operatorname{tr}\bigl(\nabla_{\xi}A^{*}_{\xi}\bigr) - \operatorname {tr}(\nabla _{\xi}A_{N}) \bigr] - \operatorname{div}^{g}\tau^{N}{^{\sharp_{\eta}}},\end{aligned} $$

*where* $r^{\eta}$ *denotes the scalar curvature of the non*-*degenerate metric* $g_{\eta}$ *on* *M*, *and* $r^{0}$ *is the extrinsic scalar curvature on the rigged null hypersurface* $(M, g, N )$ *given by* $r^{0}=g_{\eta}^{\alpha\beta}\operatorname{Ric}^{0}_{\alpha\beta}$, *with respect to a local quasi*-*orthonormal frame field* $(e_{0} := \xi, e_{1},\ldots, e_{n} )$ *for* $(M, g_{\eta})$.

### Proof

We have $r^{\eta}=g_{\eta}^{\alpha\alpha}\operatorname{Ric}^{\eta }_{\alpha \alpha}$ in a local quasi-orthonormal frame field $(e_{0}:=\xi,e_{1},\ldots,e_{n})$ for $(M,g_{\eta})$ with span $(e_{1},\ldots,e_{n})=\mathscr{S}(N)$. But

$$\begin{aligned} \operatorname{Ric}^{\eta}_{\alpha\alpha} ={}& \operatorname{Ric}^{0}_{\alpha\alpha} - \bigl[ \bigl\langle A^{\star}_{\xi}e_{\alpha},e_{\alpha} \bigr\rangle -\langle A_{N}e_{\alpha},e_{\alpha}\rangle + \tau^{N}(e_{\alpha})\eta(e_{\alpha}) \bigr]\operatorname{tr}A^{\star}_{\xi}\\ &+ \bigl[\bigl\langle \bigl(\nabla_{\xi}A^{\star}_{\xi}\bigr) (e_{\alpha}),e_{\alpha }\bigr\rangle -\bigl\langle ( \nabla_{\xi}A_{N}) (e_{\alpha}),e_{\alpha}\bigr\rangle \bigr] \\ & + \bigl[\bigl(\nabla_{\xi}\tau^{N}\bigr) (e_{\alpha})\eta(e_{\alpha}) -\bigl(\nabla_{e_{\alpha}} \tau^{N}\bigr) (e_{\alpha}) \bigr].\end{aligned} $$

Hence, by contracting each side with $g_{\eta}^{\alpha\alpha}$ and taking into account Proposition 3.1 along with the following facts: $(\nabla_{e_{i}}\tau)(e_{i})=\eta(\tau^{\sharp}) g( A^{\star}_{\xi}e_{i},e_{i}) +g( \nabla_{e_{i}}\tau^{\sharp},e_{i}) = g( \nabla_{e_{i}}\tau^{\sharp},e_{i})$, $g_{\eta}^{\alpha\alpha}(\nabla_{\xi}\tau^{N})(e_{\alpha})\eta (e_{\alpha})=0$, $g_{\eta}^{\alpha\alpha}\langle(\nabla_{\xi}A_{N})(e_{\alpha }),e_{\alpha }\rangle = \operatorname{tr}(\nabla_{\xi}A_{N})+g_{\eta}(\nabla_{\xi}(\tau ^{N\sharp _{\eta}}),\xi)$ and $g_{\eta}^{\alpha\alpha}\tau^{N}(e_{\alpha})\eta(e_{\alpha})=0, g_{\eta}^{\alpha\alpha}\langle(\nabla_{\xi}A^{\star})(e_{\alpha}),e_{\alpha }\rangle =\operatorname{tr}(\nabla_{\xi}A^{\star}_{\xi})$, we get Eq. (43). □

### Lemma 3.2

*Let* $(M,g,N) $ *be a closed normalized null hypersurface with rigged* *ξ* *and* $\tau^{N}(\xi) = 0 $ *in a Lorentzian manifold*. *Then*, *for all* $x \in M$ *and a non*-*degenerate plane* $\pi\in\mathscr{S}_{x}N$, *and* $\xi\in\Gamma (TM)^{\bot}$, *we have*

44 $$ \begin{aligned}[b]K_{\eta}(\pi)={}&K(\pi) + B^{N}(X,Y)^{2}-B^{N}(X,X)B^{N}(Y,Y) \\ &+B^{N}(X,X)C^{N}(Y,Y)-B^{N}(X,Y)C^{N}(X,Y),\end{aligned} $$

*where* *X* *and* *Y* *are orthogonal unit vectors in* $\mathscr{S}_{x} (N )$ *and* $\pi=\operatorname{Span}\{X,Y\}$.

### Proof

Suppose *π* is a non-degenerate plane (for $g_{\eta}$) in $T_{p} M$. The real number

45 $$ K_{\eta}(\pi)=\frac{ g_{\eta}(R^{\eta}(U,V)V,U)}{g_{\eta }(U,U)g_{\eta }(V,V)-(g_{\eta}(U,V))^{2}} $$

is the sectional curvature of *π* (with respect to $g_{\eta}$). Observe that a plane $\pi\subset\mathscr{S}(N)$ is both non-degenerate with respect to $g_{\eta}$ and *g* (simultaneously) or not. Now, (44) is a direct consequence of (35) in Eq. (45), taking into account the fact, without loss of generality, that we have assumed *X* and *Y* to be $g_{\eta}$-unit and orthogonal in $\mathscr{S}(N)$ (and hence also for *g*). □

## Relations between the associated Riemannian geometry and Riemannian geometry constructed on Lorentzian manifold

### Isometric immersion of a null hypersurface equipped with Riemannian associated metric

Given a null hypersurface $(M,g)$ in a Lorentzian manifold $(\overline{M}^{n+2},\overline{g})$, first, we fix a null rigging *N* fixed on *M̅*, *θ* is an 1-form given by (10). Let *ν* be an 1-form given by

46 $$ \nu(X)=\overline{g}(\xi,X), \quad X\in\Gamma(T\overline{M}), $$

with *ξ* defined along the null hypersurface, and also $\nu(N)=1$, $\nu(\xi)=0$, $\theta(N)=0$, $\theta(\xi)=1$. From now on, we suppose that Eq. (8) holds globally on $\overline{M}^{n+2}$, that is, $\overline{g}_{x}(N_{x},N_{x})=0$, $\overline{g}_{x}(N_{x},\xi_{x})=1$, $\overline{g}_{x}(\xi_{x},\xi_{x})=0$ $\forall x\in\overline{M}^{n+2}$. Now, we define the Riemannian metric $\overline{g}_{\eta}$ from the Lorentzian metric *g̅*, for all $X,Y\in\Gamma(T\overline{M})$, by

47 $$ \overline{g}_{\eta}(X,Y)= \overline{g}(X,Y)+ \bigl[ (\theta-\nu )\otimes (\theta-\nu) \bigr](X,Y). $$

For the proof, it is well known that $T\overline{M} = \Bbb{D}\otimes_{\mathrm{orth}} \mathscr{S}(N)$, where $\Bbb{D}=\operatorname{Span}\{N,\xi\}$, which means that, for all $X\in\Gamma(T\overline{M})$, one has

48 $$\begin{aligned}& X = X^{\mathscr{S}(N)} + \alpha\xi+ \beta N , \end{aligned}$$

49 $$\begin{aligned}& \left \{ \textstyle\begin{array}{l} \overline{g}(X,N)=\overline{g}(X^{\mathscr{S}(N)},N)+\alpha\overline{g}(\xi,N)+\beta\overline{g}(N,N),\\ \overline{g}(\xi,X)=\overline{g}(X^{\mathscr{S}(N)},\xi) +\alpha \overline{g}(\xi,\xi)+\beta\overline{g}(N,\xi), \end{array}\displaystyle \right . \end{aligned}$$

50 $$\begin{aligned}& \alpha=\theta(X),\qquad \beta=\nu(X). \end{aligned}$$

Putting (50) in (48), we have

51 $$ X = X^{\mathscr{S}(N)}+\theta(X) \xi +\nu(X)N $$

and

52 $$ \overline{g}_{\eta}(X,X) =\overline{g} \bigl(X^{\mathscr{S}(N)},X^{\mathscr{S}(N)}\bigr) +\theta(X)^{2}+ \nu(X)^{2}. $$

As $\Bbb{D}= \operatorname{Span}\{N_{x},\xi_{x}\}$ is a hyperbolic, it follows that the screen distribution $\mathscr{S}(N)$ on $M \subset\overline{M}^{n+2}$ is Riemannian and the induced metric on $\mathscr{S}(N)$ is positive definite,

53 $$ 0\leq\overline{g}\bigl(X^{\mathscr{S}(N)},X^{\mathscr{S}(N)}\bigr)= \overline{g}(X,X) -2\theta(X)\nu(X). $$

From (52) and (53), we have $\forall X \in\Gamma(T\overline{M})$ $\overline{g}_{\eta}(X,X)\geq0 $, and

$$ \overline{g}_{\eta}(X,X)= 0 \quad\Rightarrow\quad \left \{ \textstyle\begin{array}{l} \theta(X)^{2}=0,\\ \nu(X)^{2}=0 ,\\ \overline{g}(X^{\mathscr{S}(N)},X^{\mathscr{S}(N)})=0 \end{array}\displaystyle \right .\quad \Rightarrow\quad \left \{ \textstyle\begin{array}{l} \alpha=0,\\ \beta=0,\\ \overline{g}(X^{\mathscr{S}(N)},X^{\mathscr{S}(N)}) =0 \Rightarrow X^{\mathscr{S}(N)}= 0, \end{array}\displaystyle \right . $$

which shows that

54 $$ \overline{g}_{\eta}(X,X)=0\quad\Rightarrow\quad X=0. $$

Clearly $\overline{g}_{\eta}$ is a Riemannian metric on *M̅* and $i^{\star}{\overline{g}_{\eta}}=g_{\eta}$, which shows that the null hypersurface $M^{n+1}$ equipped with a Riemannian metric $g_{\eta}$ is isometrically immersed in a Riemannian manifold $(\overline{M}^{n+2},\overline{g_{\eta}})$.

### Link between the associated Riemannian geometry of $g_{\eta}$ and Riemannian geometry of $\overline{g}_{\eta}$

A striking fact is that the null rigging of the null hypersurface *M* is the normal unit vector field to the immersion of $(M,g_{\eta})$ into $(\overline{M}, \overline{g}_{\eta})$. Thus,

55 $$\begin{aligned}& \overline{g}_{\eta}(N,X)=0,\quad \forall X\in\Gamma(TM), \end{aligned}$$

56 $$\begin{aligned}& \overline{g}_{\eta}(N,N)=1. \end{aligned}$$

Let $\nabla^{\eta}$ and $\overline{\nabla}{^{\eta}}$ be the Levi-Civita connections with respect to $g_{\eta}$ and $\overline{g}_{\eta}$. Therefore, the Gauss–Weingarten formulae become

57 $$\begin{aligned}& \overline{\nabla}^{\eta}_{X}Y= \nabla^{\eta}_{X}Y+\alpha(X,Y)N, \end{aligned}$$

58 $$\begin{aligned}& \overline{\nabla}^{\eta}_{X}N=-A^{\eta}_{N}X. \end{aligned}$$

The *α* from Eq. (57) is rather a $(0,2)$-tensor on a line valued in $\Gamma(TM^{\bot})$. Then the following holds:

59 $$ \alpha(X,Y) = g_{\eta}\bigl(A^{\eta}_{N}X,Y \bigr),\quad \forall X,Y\in\Gamma(TM), N\in \Gamma\bigl(TM^{\bot}\bigr), $$

where $A_{N}^{\eta}$ is the shape operator of *M* with respect to *N*. For any $X\in\Gamma(TM)$, we note that the following relations are satisfied: $\overline{g}_{\eta}(N,X)=\nu(X)$, $\overline{g}_{\eta}(\xi,X)=\theta(X)$.

#### Proposition 4.1

*Let* $(M, g_{\eta} )$ *be a null hypersurface equipped with associated Riemannian metric* $g_{\eta}$ *in* $(n+2)$-*dimensional Riemannian manifold* $(\overline{M},\overline{g}_{\eta})$, $\overline{R}^{\eta}$ *and* $R^{\eta}$ *denote the Riemann curvature tensors of* $\overline{\nabla}^{\eta}$ *and* $\nabla ^{\eta}$, *respectively*. *Then*, *using the Gauss–Weingarten formulae* (57) *and* (58), *for all* $X,Y,Z,W \in\Gamma(T M )$ *and* $N\in\Gamma(TM^{\bot})$, *we prove the following*:

60 $$\begin{aligned}& \begin{aligned}[b]\overline{g}_{\eta}\bigl(\overline{R}^{\eta}(X,Y)Z,W \bigr) ={}&g_{\eta}\bigl(R^{\eta}(X,Y)Z,W\bigr)+\alpha(X,Z) \alpha(Y,W) \\ &-\alpha(Y,Z) \alpha(X,W),\end{aligned} \end{aligned}$$

61 $$\begin{aligned}& \overline{g}_{\eta}\bigl(\overline{R}^{\eta}(X,Y)Z,N \bigr)=\bigl(\nabla^{\eta }_{X}\alpha\bigr) (Y,Z)- \bigl( \nabla^{\eta}_{Y}\alpha\bigr) (X,Z). \end{aligned}$$

Let $\{ e_{0}=\xi, e_{1}, \ldots, e_{n+1}=N\}$ be any orthonormal basis for $T_{x}\overline{M}$. The sectional curvature of a plane *π* spanned by orthogonal unit vectors $e_{a}$ and $e_{b}$ is given by

62 $$ \overline{K}_{\eta}(\pi)=\overline{g}_{\eta} \bigl(\overline{R}^{\eta }(e_{a},e_{b})e_{b}, e_{a} \bigr). $$

For a fixed $a\in\{0, \ldots, n+1\}$, the Ricci curvature of $e_{a}$, is defined by

63 $$ \overline{\operatorname{Ric}}^{\eta}(e_{a})= \sum _{a\neq b=0}^{n+1}\overline{K}_{\eta}(\pi). $$

#### Theorem 4.1

*Let* $(M, g_{\eta} )$ *be a null hypersurface equipped with associated Riemannian metric* $g_{\eta}$ *in* $(n+2)$-*dimensional Riemannian manifold* $(\overline{M},\overline{g}_{\eta})$. *Then*, *for all* $X, Y, Z, W \in\Gamma(T M )$ *and* $N \in\Gamma(T M^{\bot})$, *we have*

64 $$\begin{aligned}& \overline{\operatorname{Ric}}^{\eta}(X,Y)= \operatorname{Ric}^{\eta }(X,Y)+g_{\eta}\bigl(A^{\eta}_{N}X,A^{\eta}_{N}Y \bigr)- g_{\eta}\bigl(A^{\eta}_{N}X,Y \bigr)\operatorname{tr}A^{\eta}_{N}, \end{aligned}$$

65 $$\begin{aligned}& \overline{K}^{\eta}(\pi)= K^{\eta}( \pi)+g_{\eta}\bigl(A^{\eta}_{N}X,Y\bigr)g_{\eta}\bigl(A^{\eta}_{N}Y,X\bigr) -g_{\eta}\bigl(A^{\eta}_{N}Y,Y\bigr)g_{\eta}\bigl(A^{\eta}_{N}X,X \bigr) \end{aligned}$$

*for all orthonormal unit vectors* $X,Y\in\Gamma(TM)$. *We have*

66 $$ \overline{r}^{\eta}(x)=r^{\eta}(x)+ \operatorname{tr} \bigl(\bigl(A^{\eta }_{N}\bigr)^{2} \bigr)- \bigl(\operatorname{tr}A^{\eta}_{N} \bigr)^{2}; $$

*here* $\overline{\operatorname{Ric}}^{\eta}$ *and* $\operatorname {Ric}^{\eta}$ *denote the Ricci curvature of* $\overline{\nabla}^{\eta}$ *and* $\nabla^{\eta}$, *respectively*, *and* $\overline{K}_{\eta}(\pi)$ *the sectional curvature* (*with respect to* $\overline{g}_{\eta}$), *and* $K_{\eta}(\pi)$ *the sectional curvature of* $\pi=\operatorname{Span}\{X,Y\}$ (*with respect to* $g_{\eta}$).

### Relationship between the geometry of $(\overline{M}, \overline{g}_{\eta})$ and $(\overline{M},\overline{g})$

First, we relate the Levi-Civita connection of $\overline{g}_{\eta}$ and *g̅* in the following.

#### Proposition 4.2

*Let* $(M,g)$ *be a null hypersurface of a Lorentzian manifold* $(\overline {M}^{n+2},\overline{g})$, $(\overline{M}, \overline{g}_{\eta} )$ *be a Riemannian manifold constructed from the ambient Lorentzian*, *N* *be a null rigging for* *M* *fixed on* *M̅* *satisfying* (8) *and* *ξ* *be a rigged field of* *M*. *Then*, *for all* $X,Y\in\Gamma(T \overline{M} )$, *we have*

67 $$ \begin{aligned}[b] \overline{\nabla}^{\eta}_{X}Y ={}&\overline{ \nabla}_{X}Y -\frac{1}{2} \bigl\{ (L_{N-\xi}\overline{g}) (X,Y) \bigr\} (N-\xi) \\ &+ \bigl(\theta(Y)-\nu(Y) \bigr) \bigl[i_{X} (d\theta-d\nu ) \bigr]^{\sharp_{\overline{g}_{\eta}}} \\ &+ \bigl(\theta(X)-\nu(X) \bigr) \bigl[i_{Y} (d\theta-d\nu ) \bigr]^{\sharp _{\overline{g}_{\eta}}}.\end{aligned} $$

*In particular*, *for all* $X,Y\in(TM)\subset i^{\star}(T\overline{M} )$, *with* *θ* *closed*, *we have*

68 $$ \overline{\nabla}^{\eta}_{X}Y=\overline{ \nabla}_{X}Y -\frac{1}{2} \bigl\{ (L_{N-\xi}\overline{g}) (X,Y) \bigr\} (N-\xi). $$

#### Proof

As the connections $\overline{\nabla}^{\eta}$ and ∇̅ are symmetric, we can write

$$ \overline{\nabla}^{\eta}_{X}Y =\overline{ \nabla}_{X}Y+D^{\eta}(X,Y),\quad\forall X,Y\in\Gamma(TM). $$

Using the fact that $D^{\eta}$ is a symmetric tensor and $\overline{\nabla}^{\eta}$ is a $\overline{g}_{\eta}$-metric connection, we have

$$\begin{aligned} \overline{g}_{\eta}\bigl( D^{\eta}(X,Y),Z \bigr) +\overline{g}_{\eta}\bigl(Y,D^{\eta}(X,Z) \bigr) ={}& (\overline{\nabla}_{X}\overline{g}_{\eta}) (Y,Z) \\ ={}& \bigl[ (\overline{\nabla}_{X}\theta) (Y)-(\overline{\nabla}_{X}\nu ) (Y) \bigr]\theta(Z) \\ &+ \bigl[ (\overline{\nabla}_{X}\theta) (Z)-(\overline{\nabla}_{X}\nu ) (Z) \bigr]\theta(Y) \\ &- \bigl[ (\overline{\nabla}_{X}\theta) (Z)-(\overline{\nabla}_{X}\nu ) (Z) \bigr]\nu (Y) \\ &- \bigl[ (\overline{\nabla}_{X}\theta) (Y)-(\overline{\nabla}_{X}\nu ) (Y) \bigr]\nu(Z).\end{aligned} $$

By a circular permutation, we get similar expressions for $\overline{g}_{\eta}( D^{\eta}(Y,Z),X )+\overline{g}_{\eta}(Z,D^{\eta}(Y,X) )$ and $\overline{g}_{\eta}( D^{\eta}(Z,X),Y ) +\overline{g}_{\eta}(X,D^{\eta}(Z,Y) )$. Summing the first two expressions minus the last one leads to

$$\begin{aligned}& \begin{aligned} 2\overline{g}_{\eta}\bigl(D^{\eta}(X,Y),Z \bigr) ={}& \bigl[ (L_{N}\overline{g}) (X,Y)-(L_{\xi}\overline{g}) (X,Y)\bigr] \overline{g}(N,Z) \\ &- \bigl[ (L_{N}\overline{g}) (X,Y)-(L_{\xi}\overline{g}) (X,Y) \bigr]\overline{g}(\xi,Z) \\ &+ 2\theta(Y) \bigl[d\theta(X,Z) -d\nu(X,Z) \bigr] \\ &-2\nu(Y) \bigl[d\theta(X,Z) -d\nu(X,Z) \bigr] \\ &+2\theta(X) \bigl[d\theta(Y,Z) -d\nu(Y,Z) \bigr] \\ &-2\nu(X) \bigl[d\theta(Y,Z)-d\nu(Y,Z) \bigr],\end{aligned} \\& \begin{aligned}2\overline{g}_{\eta}\bigl(D^{\eta}(X,Y),Z \bigr) ={}& \bigl[ (L_{N}\overline{g}) (X,Y)-(L_{\xi}\overline{g}) (X,Y) \bigr] \overline{g}_{\eta}(\xi,Z) \\ &- \bigl[ (L_{N}\overline{g}) (X,Y)-(L_{\xi}\overline{g}) (X,Y) \bigr]\overline{g}_{\eta}(N,Z) \\ &+2\theta(Y) \bigl[\overline{g}_{\eta}\bigl(\bigl(i_{X}(d \theta-d\nu )\bigr)^{\sharp _{\overline{g}_{\eta}}},Z \bigr) \bigr] \\ &-2\nu(Y) \bigl[\overline{g}_{\eta}\bigl(\bigl(i_{X}(d \theta-d\nu)\bigr)^{\sharp _{\overline {g}_{\eta}}},Z\bigr) \bigr] \\ &+2\theta(X) \bigl[\overline{g}_{\eta}\bigl(\bigl(i_{Y}(d \theta-d\nu )\bigr)^{\sharp _{\overline{g}_{\eta}}},Z \bigr) \bigr] \\ &-2\nu(X) \bigl[\overline{g}_{\eta}\bigl(\bigl(i_{Y}(d \theta-d\nu)\bigr)^{\sharp _{\overline{g}_{\eta}}},Z \bigr) \bigr].\end{aligned} \end{aligned}$$

With

$$\begin{gathered} (L_{N}\overline{g}) (X,Y)= (\overline{\nabla}_{X}\theta) (Y)+ (\overline{\nabla}_{Y}\theta) (X), \\ (L_{\xi}\overline{g}) (X,Y)= (\overline{\nabla}_{X}\nu) (Y)+ ( \overline{\nabla}_{Y}\nu) (X), \\ 2(d\theta) (X,Y) = (\overline{\nabla}_{X}\theta) (Y)-(\overline{\nabla}_{Y}\theta) (X).\end{gathered} $$

It follows that

$$\begin{aligned} D^{\eta}(X,Y) ={}& {-}\frac{1}{2} \bigl\{ (L_{N-\xi}\overline{g}) (X,Y) \bigr\} (N-\xi) + \bigl(\theta(Y)-\nu(Y) \bigr) \bigl[\bigl(i_{X}(d\theta-d\nu) \bigr)^{\sharp_{\overline{g}_{\eta}}} \bigr] \\ &+ \bigl(\theta(X)-\nu(X) \bigr) { \bigl[\bigl(i_{Y}(d\theta-d\nu) \bigr)^{\sharp_{\overline{g}_{\eta}}} \bigr].}\end{aligned} $$

 □

#### Remark 4.1

$\forall X,Y \in\Gamma(TM)$, $\nu(X) = 0 $, and if *θ* is closed, we have

69 $$ \overline{\nabla}^{\eta}_{X}Y =\overline{ \nabla}_{X}Y -\frac{1}{2} \bigl\{ (L_{N-\xi}\overline{g}) (X,Y) \bigr\} (N-\xi). $$

For a proper conformal Killing field $N-\xi$, with conformal factor 2*λ* i.e. $L_{N-\xi}\overline{g} = 2\lambda\overline{g} $ and $\lambda= \frac{1}{n+2}\operatorname{div}^{\overline{g}}(N-\xi)$, we have

70 $$ D^{\eta}(X,Y) = -\lambda\overline{g}(X,Y) (N-\xi) $$

and

71 $$ \overline{\nabla}^{\eta}_{X}Y = \overline{\nabla}_{X}Y-\lambda\overline{g}(X,Y) (N-\xi). $$

From now on, throughout the paper, assume $N-\xi$, to be proper conformal Killing with conformal factor 2*λ* with respect to the metric *g̅*.

#### Lemma 4.1

*Let* $(M^{n+1},g,N)$ *be a closed normalized null hypersurface in Lorentzian manifold* $(\overline{M},\overline{g})$, $(N-\xi)$ *be a proper conformal Killing with conformal factor* 2*λ* *and* $\lambda= \frac{1}{n+2}\operatorname{div}^{\overline{g}}(N-\xi)$ *and* $\tau^{N}(\xi)=0$. *Then*

72 $$ A^{\eta}_{N} = A_{N}- \tau^{N}(\cdot)N+\lambda\eta(\cdot) (N-\xi). $$

#### Proof

Putting $Y=N$ in (71), we have

73 $$ \overline{\nabla}^{\eta}_{X}N = \overline{\nabla}_{X}N-\lambda\overline{g}(X,N) (N-\xi). $$

By applying (13) and (58) in (73), we have the desired result. □

#### Theorem 4.2

*Let* $\overline{R}^{\eta}$ *and* *R̅* *denote the Riemann curvatures tensors of* $\overline{\nabla}^{\eta}$ *and* ∇̅, *respectively*. *Then*, *for all* $X, Y, Z \in\Gamma(T M ) \subset i^{\star}(T\overline{M} )$, $\xi\in\Gamma(T M^{\bot})$, *N* *be a null rigging for* *M* *fixed on* *M̅* *and* $N-\xi$ *a proper conformal Killing field with* *θ* *closed*, *the following holds*:

74 $$ \begin{aligned}[b] \overline{g}_{\eta}\bigl(\overline{R}^{\eta}(X,Y)Z,W \bigr) ={}&\overline{g}\bigl(\overline{R}(X,Y)Z,W\bigr) +\eta(W) \bigl[ \bigl(R^{0}(X,Y)Z\cdot\lambda\bigr) -\bigl(\nabla_{X}B^{N} \bigr) (Y,Z) \\ & +\bigl(\nabla_{Y}B^{N}\bigr) (X,Z)-\tau^{N}(X)B^{N}(Y,Z)+ \tau^{N}(Y)B^{N}(X,Z) \\ &+\bigl(\nabla_{X}C^{N}\bigr) (Y,PZ) -\bigl( \nabla_{Y}C^{N}\bigr) (X,PZ)-2\,d\tau^{N}(X,Y)\eta(Z) \\ &+\tau^{N}(Y)C^{N}(X,PZ)-\tau^{N}(X)C^{N}(Y,PZ) \bigr] \\ &+\lambda\overline{g}(Y,Z) \bigl[C^{N}(X,PW)-B^{N}(X,W)\\&- \tau^{N}(X)\eta (W)-\lambda\eta(X)\eta(W) \bigr] \\ &-\lambda\overline{g}(X,Z) \bigl[C^{N}(Y,PW)-B^{N}(Y,W)\\&- \tau^{N}(Y)\eta (W)-\lambda\eta(Y)\eta(W) \bigr]\end{aligned} $$

*for all* $X,Y,Z,W\in\Gamma(TM)$. *Here* $R^{0}(X,Y)Z\cdot\lambda= X(\lambda)\overline{g}(Y,Z)-Y(\lambda) \overline{g}(X,Z)$.

#### Proof

The curvature tensor field of $\overline{\nabla}^{\eta}$ is defined by

75 $$ \overline{R}^{\eta}(X,Y)Z = \bigl[\overline{\nabla}^{\eta}_{X},\overline{\nabla}^{\eta}_{Y} \bigr]Z -\overline{\nabla}^{\eta}_{[X,Y]}Z. $$

By application of (71) in (75), we get (74). □

#### Theorem 4.3

*Let* $\overline{\operatorname{Ric}}^{\eta}$ *and* $\overline {\operatorname{Ric}}$ *be the Ricci curvatures of* $\overline{\nabla}^{\eta }$ *and* ∇̅, *respectively*, *then let*, *for all* $X, Y, Z \in \Gamma(T M ) \subset i^{\star}(T\overline{M} )$, $\xi\in T M^{\bot}$ *and* *N* *be a null rigging for* *M* *fixed on* *M̅*, *and* $N-\xi$ *a proper conformal Killing field with* *θ* *closed*, *then the following holds*:

76 $$ \begin{aligned}[b] \overline{\operatorname{Ric}}^{\eta}(X,Y)={}& \overline{ \operatorname {Ric}}(X,Y)-R^{0}(N,X)Y\cdot\lambda +\bigl( \nabla_{X}\tau^{N}\bigr) (Y)+\bigl\langle ( \nabla_{\xi}A_{N}) (X),Y\bigr\rangle \\ &-\bigl(\nabla_{\xi}\tau^{N}\bigr) (X)\eta(Y)-\bigl\langle A_{\xi}^{\star}X,A_{N}Y\bigr\rangle +\bigl( \nabla_{Y}\tau^{N}\bigr) (X)+\bigl\langle ( \nabla_{\xi}A_{N}) (Y),X\bigr\rangle \\ &-\bigl(\nabla_{\xi}\tau^{N}\bigr) (Y)\eta(X)-\bigl\langle A_{\xi}^{\star}Y,A_{N}X\bigr\rangle -\lambda\bigl[ \langle A_{N}X,Y\rangle-\bigl\langle A^{\star}_{\xi}X,Y \bigr\rangle \bigr] \\ &+\bigl[\xi(\lambda)-\lambda^{2} +\lambda\bigl(\operatorname{tr}A_{N}-\operatorname{tr}A_{\xi}^{\star}\bigr)\bigr]\langle X,Y\rangle +\lambda\eta(Y)\bigl[\tau(X)-\lambda\eta(X)\bigr].\end{aligned} $$

#### Proof

Let $x\in\overline{M}$ and $(e_{0}=\xi, e_{1},\ldots, e_{n},e_{n}+1=N)$ be a quasi-orthonormal basis for $(T_{x}\overline{M}, \overline{g}_{x})$ with span $(e_{1}, \ldots, e_{n})=\mathscr{S}(N)_{|_{p}}$. Suppose that the indices *i*, *j*, *k* run over the range $1,\ldots,n$, $\alpha,\beta,\gamma,\ldots\in\{0,\ldots,n\}$ and $a,b,\ldots ,\in\{ 0,\ldots,n+1\}$; then

77 $$ \begin{aligned}[b]\overline{\operatorname{Ric}}^{\eta}(X,Y) &= \operatorname{trace} \bigl(Z\mapsto\overline{R}^{\eta}(Z,X)Y \bigr) \\ &= \sum_{a=o}^{n+1}\overline{g}^{aa}_{\eta} \overline{g}_{\eta} \bigl(\overline{R}^{\eta}(e_{a},X)Y,e_{a}\bigr) \\ &= \overline{g}_{\eta} \bigl(\overline{R}^{\eta}(\xi,X)Y,\xi \bigr)+ \sum_{i=1}^{n}\overline{g}^{ii}_{\eta} \overline{g}_{\eta} \bigl(\overline{R}^{\eta}(e_{i},X)Y,e_{i}\bigr)+\overline{g}_{\eta} \bigl(\overline{R}^{\eta}(N,X)Y,N\bigr).\end{aligned} $$

From (74), with $\overline{g}_{\eta}(N-\xi,\xi)=-1$, $\overline{g}_{\eta}(N-\xi,N)=1$, and $\overline{g}_{\eta}(A_{N}X,e_{i})= \overline{g}(A_{N}X,e_{i})$, we get

78 $$\begin{aligned}& \overline{g}_{\eta} \bigl(\overline{R}^{\eta}( \xi,X)Y,\xi\bigr)= \overline{g}_{\eta} \bigl(\overline{R}(\xi,X)Y,\xi \bigr)- \bigl[\xi(\lambda)\overline{g}(X,Y)-X(\lambda)\overline{g}(\xi ,Y)\bigr] \overline{g}_{\eta}(N-\xi,\xi) \\& \phantom{\overline{g}_{\eta} \bigl(\overline{R}^{\eta}( \xi,X)Y,\xi\bigr) ) =}-\lambda\overline{g}(X,Y)\bigl[-\overline{g}_{\eta}(A_{N}\xi, \xi)+\tau ^{N}(\xi )\overline{g}_{\eta}(N,\xi) +\overline{g}_{\eta}\bigl(A^{\star}_{\xi}\xi,\xi\bigr) \\& \phantom{\overline{g}_{\eta} \bigl(\overline{R}^{\eta}( \xi,X)Y,\xi\bigr) =}+\tau^{N}(\xi)\overline{g}_{\eta}(\xi,\xi) -\lambda\eta(\xi) \overline{g}_{\eta}(N-\xi,\xi)\bigr] +\lambda\overline{g}(\xi,Y)\bigl[- \overline{g}_{\eta}(A_{N}X,\xi) \\& \phantom{\overline{g}_{\eta} \bigl(\overline{R}^{\eta}( \xi,X)Y,\xi\bigr) ) =}+\tau^{N}(X)\overline{g}_{\eta}(N,\xi)+\overline{g}_{\eta}\bigl(A^{\star}_{\xi}X,\xi\bigr) + \tau^{N}(X)\overline{g}_{\eta}(\xi,\xi) \\& \phantom{\overline{g}_{\eta} \bigl(\overline{R}^{\eta}( \xi,X)Y,\xi\bigr) ) =}-\lambda\eta(X)\overline{g}_{\eta}(N-\xi,\xi)\bigr] \\& \phantom{\overline{g}_{\eta} \bigl(\overline{R}^{\eta}( \xi,X)Y,\xi\bigr) ) }=\bigl(\nabla_{X}\tau^{N}\bigr) (Y)+ g \bigl(( \nabla_{\xi}A_{N}) (X),Y \bigr)-\bigl(\nabla _{\xi}\tau^{N}\bigr) (X)\eta(Y) \\& \phantom{\overline{g}_{\eta} \bigl(\overline{R}^{\eta}( \xi,X)Y,\xi\bigr) ) =}-g\bigl(A^{\star}_{\xi}X,A_{N}Y\bigr) +\bigl[\xi( \lambda)-\lambda^{2}\bigr]g(X,Y), \end{aligned}$$

79 $$\begin{aligned}& \overline{g}_{\eta} \bigl(\overline{R}^{\eta}(e_{i},X)Y,e_{i} \bigr) = \overline{g}_{\eta} \bigl(\overline{R}(e_{i},X)Y,e_{i} \bigr)- \bigl[e_{i}(\lambda)\overline{g}(X,Y)-X(\lambda)\overline{g}(e_{i},Y)\bigr]\overline{g}_{\eta}(N-\xi,e_{i}) \\& \phantom{\overline{g}_{\eta} \bigl(\overline{R}^{\eta}(e_{i},X)Y,e_{i} \bigr) ) =}-\lambda\overline{g}(X,Y)\bigl[-\overline{g}_{\eta}(A_{N}e_{i},e_{i})+ \tau ^{N}(e_{i})\overline{g}_{\eta}(N,e_{i}) +\overline{g}_{\eta}\bigl(A^{\star}_{\xi}e_{i},e_{i}\bigr) \\& \phantom{\overline{g}_{\eta} \bigl(\overline{R}^{\eta}(e_{i},X)Y,e_{i} \bigr) =}+\tau^{N}(e_{i})\overline{g}_{\eta}( \xi,e_{i}) -\lambda\eta(e_{i})\overline{g}_{\eta}(N- \xi,e_{i})\bigr] \\& \phantom{\overline{g}_{\eta} \bigl(\overline{R}^{\eta}(e_{i},X)Y,e_{i} \bigr) =}+\lambda\overline{g}(e_{i},Y)\bigl[-\overline{g}_{\eta}(A_{N}X,e_{i}) +\tau^{N}(X)\overline{g}_{\eta}(N,e_{i}) \\& \phantom{\overline{g}_{\eta} \bigl(\overline{R}^{\eta}(e_{i},X)Y,e_{i} \bigr) ) =}+\overline{g}_{\eta}\bigl(A^{\star}_{\xi}X,e_{i}\bigr)+\tau^{N}(X)\overline{g}_{\eta}( \xi,e_{i}) -\lambda\eta(X)\overline{g}_{\eta}(N- \xi,e_{i})\bigr] \\& \phantom{\overline{g}_{\eta} \bigl(\overline{R}^{\eta}(e_{i},X)Y,e_{i} \bigr) }= \overline{g} \bigl(\overline{R}(e_{i},X)Y,e_{i}\bigr) ) +\lambda\overline{g}(X,Y)\bigl[\overline{g}(A_{N}e_{i},e_{i})- \overline{g} \bigl(A^{\star }_{\xi}e_{i},e_{i} \bigr)\bigr] \\& \phantom{\overline{g}_{\eta} \bigl(\overline{R}^{\eta}(e_{i},X)Y,e_{i} \bigr) =} -\lambda\overline{g}(e_{i},Y)\bigl[\overline{g}(A_{N}X,e_{i})- \overline{g}\bigl(A^{\star}_{\xi}X,e_{i}\bigr)\bigr], \end{aligned}$$

and

80 $$ \begin{aligned}[b]\overline{g}_{\eta} \bigl(\overline{R}^{\eta}(N,X)Y,N \bigr)={}&\bigl(\nabla_{Y}\tau^{N}\bigr) (X)+ g \bigl(( \nabla_{\xi}A_{N}) (Y),X \bigr)-\bigl(\nabla _{\xi}\tau^{N}\bigr) (Y)\eta(X) \\ &-g\bigl(A^{\star}_{\xi}Y,A_{N}X\bigr) -\bigl[N( \lambda)\overline{g}(X,Y) -X(\lambda)\overline{g}(N,Y)\bigr] \\ &+\lambda\overline{g}(N,Y)\bigl[\tau^{N}(X)-\lambda\eta(X)\bigr].\end{aligned} $$

By substituting (78), (79) and (80) in (77), we have (76). □

We have a formula relating the scalar curvature $\overline{r}^{\eta}$ and *r̅* of the $\overline{g}_{\eta}$ and *g̅* in $x\in M $ as follows.

#### Theorem 4.4

81 $$ \begin{aligned}[b]\overline{r}^{\eta}(x) ={}& \overline{r}(x) + \operatorname{div}^{\overline{g}_{\eta}}\tau ^{\sharp }+\operatorname{tr}( \nabla_{\xi}A_{N})-\operatorname{tr}\bigl(A^{\star}_{\xi}A_{N}\bigr) \\ &-\lambda^{2}+\xi(\lambda)-N(\lambda).\end{aligned} $$

Let *π* be a non-degenerate plane (for $\overline{g}_{\eta}$), we define the sectional curvature of *π* (with respect to $\overline{g}_{\eta}$) in $T_{p} M$ by

82 $$ \overline{K}_{\eta}(\pi)= \frac{ \overline{g}_{\eta}(\overline{R}^{\eta}(X,Y)Y,X)}{ \overline{g}_{\eta}(X,X)\overline{g}_{\eta}(Y,Y)-(\overline{g}_{\eta}(X,Y))^{2}},\quad \forall X,Y\in \Gamma(TM)\subset i^{\star}(T\overline{M}). $$

We give now the relation between $\overline{K}_{\eta}(\pi)$ and $\overline{K}(\pi)$.

#### Theorem 4.5

*For any orthonormal vectors* $X,Y \in\Gamma(TM)\subset i^{\star}\Gamma (T\overline{M})$ *with respect to* $\overline{g}_{\eta}$, $\pi=\operatorname{Span}\{ X,Y \}$, $\xi\in\Gamma(T M^{\bot})$, *N* *be a null rigging for* *M* *fixed on* *M̅*, $N-\xi$ *a proper conformal Killing field with* *θ* *closed*,

83 $$ \begin{aligned}[b] \overline{K}_{\eta}(\pi) ={}& \bigl[1-\eta(X)^{2}- \eta(Y)^{2}\bigr]\overline{K}(\pi)+ \eta(X) \bigl[\bigl(R^{0}(X,Y)Y \bigr)\cdot\lambda- \bigl(\nabla_{X}B^{N}\bigr) (Y,Y) \\ & +\bigl(\nabla_{Y}B^{N}\bigr) (X,Y)+B^{N}(X,Y) \tau^{N}(Y)-B^{N}(Y,Y)\tau^{N}(X) \\ &+\bigl(\nabla_{X}C^{N}\bigr) (Y,PY)-\bigl( \nabla_{Y}C^{N}\bigr) (X,PY)-2\,d\tau^{N}(X,Y)\eta (Y) \\ &+\tau^{N}(Y)C^{N}(X,PY)-\tau^{N}(X)C^{N}(Y,PY) \bigr]-\lambda\eta^{2}(X) \\ &+\lambda \bigl(1-\eta^{2}(Y) \bigr) \bigl[ C^{N}(X,PX)-B^{N}(X,X)- \tau^{N}(X)\eta(X) \bigr] \\ &+\lambda\eta(X)\eta(Y) \bigl[C^{N}(Y,PX)-B^{N}(Y,X)- \tau^{N}(Y)\eta (X) \bigr].\end{aligned} $$

#### Proof

From $\overline{g}_{\eta}$-orthonormal vectors $X,Y\in\Gamma(TM) \subset i^{\star}\Gamma(T\overline{M})$ and $\pi=\operatorname{Span}\{X,Y\}$, we have $\overline{g}_{\eta}(X,Y)= 1 $ and $\overline{g}_{\eta}(X,Y)=0$. It follows with (82) that

84 $$ \overline{K}_{\eta}(\pi) =\overline{g}_{\eta}\bigl(\overline{R}^{\eta}(X,Y)Y,X\bigr). $$

Using Theorem 4.2, we get

85 $$\begin{aligned}[b] \overline{K}_{\eta}(\pi) ={}&\overline{g}_{\eta}\bigl(\overline{R}(X,Y)Y,X\bigr) -\bigl[X(\lambda)\overline{g}(Y,Y)-Y(\lambda)\overline{g}(X,Y) \bigr]\overline{g}_{\eta}(N-\xi,X) \\ &-\lambda\overline{g}(Y,Y)\bigl[-\overline{g}_{\eta}(A_{N}X,X)+ \tau ^{N}(X)\overline{g}_{\eta}(N,X) +\overline{g}_{\eta}\bigl(A^{\star}_{\xi}X,X\bigr) \\ &+\tau^{N}(X)\overline{g}_{\eta}(\xi,X) -\lambda\eta(X) \overline{g}_{\eta}(N-\xi,X)\bigr] +\lambda\overline{g}(X,Y)\bigl[ - \overline{g}_{\eta}(A_{N}Y,X) \\ &+\tau^{N}(Y)\overline{g}_{\eta}(N,X) + \overline{g}_{\eta}\bigl(A^{\star}_{\xi}Y,X\bigr) + \tau^{N}(Y)\overline{g}_{\eta}(\xi, X)-\lambda\eta(Y)\overline{g}_{\eta}(N-\xi ,X)\bigr] \\ ={}&\overline{g}\bigl(\overline{R}(X,Y)Y,X\bigr) -\theta(X)\bigl[\overline{g}\bigl( \xi,\overline{R}(X,Y)Y\bigr) - \overline{g}\bigl(N,\overline{R}(X,Y)Y\bigr)\bigr] \\ &-\bigl[\bigl(R^{0}(X,Y)Y\bigr)\cdot\lambda\bigr]\overline{g}_{\eta}(N-\xi,X) \\ &-\lambda\overline{g}(Y,Y)\bigl[-\overline{g}_{\eta}(A_{N}X,X)+ \tau ^{N}(X)\overline{g}_{\eta}(N,X) +\overline{g}_{\eta}\bigl(A^{\star}_{\xi}X,X\bigr) \\ &+\tau^{N}(X)\overline{g}_{\eta}(\xi,X) -\lambda\eta(X) \overline{g}_{\eta}(N-\xi,X)\bigr] +\lambda\overline{g}(X,Y)\bigl[ - \overline{g}_{\eta}(A_{N}Y,X) \\ &+\tau^{N}(Y)\overline{g}_{\eta}(N,X) + \overline{g}_{\eta}\bigl(A^{\star}_{\xi}Y,X\bigr) + \tau^{N}(Y)\overline{g}_{\eta}(\xi, X)-\lambda\eta(Y)\overline{g}_{\eta}(N-\xi ,X)\bigr].\end{aligned} $$

We take into account the fact that we obtain

86 $$ \begin{aligned}[b]\overline{g}\bigl(\overline{R}(X,Y)N,Y\bigr) ={}& {-}\overline{g}( \nabla_{X}A_{N}) (Y),Y) +\overline{g}(\nabla_{Y}A_{N}) (X), Y)-B^{N}(X,A_{N}Y)\eta(Y) \\ &+2 \,d\tau^{N}(X,Y)\eta(Y) -\tau^{N}(Y)\overline{g}(A_{N}X,Y) \\ &+B^{N}(Y,A_{N}X)\eta(Y) +\tau^{N}(X)\overline{g}(A_{N}Y,Y)\end{aligned} $$

and

87 $$ \overline{g}_{\eta}(N-\xi,X)= -\theta(X),\qquad \overline{g}_{\eta}(\xi,X)= \theta(X),\qquad \overline{g}_{\eta}(A_{N}X,X)= \overline{g}(A_{N}X,X). $$

Putting (23), (86), (87) in (85), we have

$$\begin{aligned}[b] \overline{K}_{\eta}(\pi) ={}& \bigl[1-\eta^{2}(X)-\eta^{2}(Y)\bigr]\overline{K}(\pi) - \eta(X)\bigl[\bigl(\nabla_{X}B^{N}\bigr) (Y,Y)-\bigl( \nabla_{Y}B^{N}\bigr) (X,Y) \\ &+\tau^{N}(Y)B^{N}(X,Y)-\tau^{N}(X)B^{N}(Y,Y) -\overline{g}\bigl(\bigl(\nabla_{X} C^{N}\bigr) (Y),Y\bigr) \\ &+B^{N}(X,A_{N}Y)\eta(Y)+ \bigl(\nabla_{Y}C^{N} \bigr) (X), Y)-B^{N}(Y,A_{N}X)\eta(Y) \\ &-B^{N}(X,A_{N}Y)\eta(Y)+ 2\,d\tau^{N}(X,Y)\eta(Y) -\tau^{N}(Y)\overline{g}(A_{N}X,Y) \\ & +B^{N}(Y,A_{N}X)\eta(Y)+\tau^{N}(X)\overline{g}(A_{N}Y,Y)-R^{0}(X,Y)Y\cdot\lambda \bigr] \\ &-\lambda\overline{g}(Y,Y)\bigl[-\overline{g}(A_{N}X,X)+\overline{g} \bigl(A^{\star}_{\xi}X,X\bigr) +\tau^{N}(X)\theta(X)+ \lambda\eta(X)\eta(X)\bigr] \\ &+\lambda\overline{g}(X,Y)\bigl[-\overline{g}(A_{N}Y,X) + \overline{g} \bigl(A^{\star}_{\xi}Y,X\bigr)+\tau^{N}(Y)\eta(X)+ \lambda\eta(Y)\eta (X) \bigr].\end{aligned} $$

With $\overline{g}((\nabla_{X}A_{N})(Y),Y) = (\nabla_{X}C^{N})(Y,PY)-B^{N}(X,A_{N}Y)\eta(Y)$, $\overline{g}(X,X) = 1-\eta^{2}(X)$ and $\overline{g}(X,Y) = \eta(X)\eta(Y)$ □

#### Corollary 4.1

*If the ambient manifold is of a constant sectional curvature* *k*, *then we have*

88 $$\begin{aligned}[b] \overline{K}_{\eta}(\pi) ={}& \bigl[1-\eta(X)^{2}- \eta(Y)^{2}\bigr]k+\eta(X) \bigl[\bigl(R^{0}(X,Y)Y\bigr). \lambda \\ &+\bigl(\nabla_{X}C^{N}\bigr) (Y,PY)-\bigl( \nabla_{Y}C^{N}\bigr) (X,PY)-2\,d\tau^{N}(X,Y)\eta (Y) \\ &+\tau^{N}(Y)C^{N}(X,PY)-\tau^{N}(X)C^{N}(Y,PY) \bigr]-\lambda\eta^{2}(X) \\ &+\lambda \bigl(1-\eta^{2}(Y) \bigr) \bigl[ C^{N}(X,PX)-B^{N}(X,X)- \tau^{N}(X)\eta(X) \bigr] \\ &+\lambda\eta(X)\eta(Y) \bigl[C^{N}(Y,PX)-B^{N}(Y,X)- \tau^{N}(Y)\eta (X) \bigr].\end{aligned} $$

#### Proof

Using the Gauss–Codazzi equation (23) if the ambient manifold has constant sectional curvature *k*, then we can write $(\nabla_{X}B^{N})(Y,Y)-(\nabla_{Y}B^{N})(X,Y)= B^{N}(Y,Y)\tau ^{N}(X)-B^{N}(X,Y)\tau^{N}(Y)$, and substituting this in (83), we have the desired result. □

#### Corollary 4.2

*For all* $X,Y\in\mathscr{S}(N)$,

89 $$ \overline{K}_{\eta}(\pi)=\overline{K}(\pi)+\lambda \bigl[C^{N}(X,PX)-B^{N}(X,X)\bigr]. $$

## Chen–Ricci inequality

In this section, we establish some basic inequalities between intrinsic invariants namely the Ricci curvature Ric, scalar curvature, shape operator $A_{N}$, Chen invariant and the extrinsic invariant called the squared mean curvature for null hypersurface $(M,g)$ in $(n+2)$-dimensional Lorentzian manifold $(\overline{M},\overline{g})$.

### Theorem 5.1

*Let* $(M,g,N )$ *be a closed normalized null hypersurface in a* $(n+2)$-*dimensional Lorentzian manifold* $(\overline{M},\overline{g})$, $N-\xi$ *be a proper conformally Killing field with conformal factor* 2*λ* *and* $\lambda=\frac {1}{n+2}\operatorname{div}^{\overline{g}}(N-\xi)$. *Then*, *for all* $X\in\mathscr{S}^{0}_{x}(1)$, *the following holds*:

90 $$ \begin{aligned}[b] \operatorname{Ric}(X) \leq{}& \overline{\operatorname{Ric}}_{T_{x}M}(X)+ \frac{1}{4}(n+1)^{2} \Vert H \Vert _{\overline{g}_{\eta}}^{2} -\bigl(R^{0}(N,X)X\bigr)\cdot\lambda-2\bigl\langle A^{\star}_{\xi}X, A_{N}X\bigr\rangle \\ &-\lambda \bigl[ \langle A_{N}X,X\rangle -\bigl\langle A^{\star}_{\xi}X, X\bigr\rangle \bigr] + \bigl\{ \xi(\lambda) - \lambda^{2} +\lambda\bigl[\operatorname{tr}A_{N} -\operatorname{tr}A^{\star}_{\xi}\bigr] \bigr\} \langle X,X\rangle \\ &+\lambda\eta(X) \bigl[ \tau^{N}(X)-\lambda\eta(X) \bigr]+ \bigl[ \bigl\langle A^{\star}_{\xi}X,X\bigr\rangle -\langle A_{N}X,X\rangle+\tau^{N}(X)\eta(X) \bigr]\operatorname{tr}A^{\star}_{\xi}\\ &-\bigl\langle \bigl(\nabla_{\xi}A_{\xi}^{\star}\bigr) (X),X\bigr\rangle + 3 \bigl[ \bigl(\nabla _{X}\tau^{N}\bigr) (X) +\bigl\langle (\nabla_{\xi}A_{N}) (X),X\bigr\rangle -\bigl( \nabla_{\xi}\tau^{N}\bigr) (X)\eta (X) \bigr],\end{aligned} $$

*where* $\overline{\operatorname{Ric}}_{(T_{x} M)}(X) $ *is the Ricci curvature of* *X* *at* *x* *with respect to* $(\overline{M}, \overline{g})$, $\lambda=\frac {1}{n+2}\operatorname{div}^{\overline{g}}(N-\xi)$ *and the mean curvature vector* $H(x) $ *is given by* $H(x)=\frac {1}{n+1}(\operatorname{tr} A^{\eta}_{N})N_{x}$.

*The equality case of* (90) *is satisfied by* $X\in \mathscr {S}^{0}_{x}(1)$ *if and only if*

91 $$ \left \{ \textstyle\begin{array}{l} \alpha(X,Y)=0,\quad\forall Y\in TM, \qquad g_{\eta}(X,Y)= 0,\\ 2\alpha(X,X)=(n+1)H, \quad\forall Y\in O_{\eta}(X). \end{array}\displaystyle \right . $$

*The equality case of* (90) *holds for all unit vectors* $X\in\mathscr{S}^{0}_{x}(1)$ *and for all* $x \in M $ *if and only if either* *M* *is totally geodesic or* $n=1$.

### Proof

The null hypersurface equipped with Riemannian metric $g_{\eta}$ is a Riemannian hypersurface, and as $(M, g_{\eta})$ is isometrically immersed in a Riemannian manifold $(\overline{M},\overline{g}_{\eta})$, using standard techniques as [19], we have this inequality:

92 $$ \operatorname{Ric}^{\eta}(X)\leq\overline{\operatorname {Ric}}^{\eta }_{(T_{x}M)}(X) + \frac{1}{4}(n+1)^{2} \Vert H \Vert _{\overline{g}_{\eta}}^{2}, $$

where $X\in\mathscr{S}^{0}_{x}(1)$. If we put the equality (39) of Theorem 3.1 in (92), we get

93 $$ \begin{aligned}[b]\operatorname{Ric}(X) \leq{}& \overline{\operatorname{Ric}}^{\eta}_{T_{x}M}(X)+ \frac {1}{4}(n+1)^{2} \Vert H \Vert _{\overline{g}_{\eta}}^{2} + \bigl[\bigl\langle A^{*}_{\xi}X,X\bigr\rangle - \langle A_{N}X,X\rangle \\ &+ \tau^{N}(X)\eta(X) \bigr]\operatorname{tr}A^{*}_{\xi}-\bigl\langle \bigl( \nabla_{\xi}A^{*}_{\xi}\bigr) (X),X \bigr\rangle +\bigl\langle ( \nabla_{\xi}A_{N}) (X),X \bigr\rangle \\ &-\bigl(\nabla_{\xi}\tau^{N}\bigr) (X)\eta(X) + \bigl( \nabla_{X}\tau^{N}\bigr) (X);\end{aligned} $$

with (76) plugged into (93), we have the inequality (90) □

### Corollary 5.1

*Let* $(M,g,N )$ *be a closed normalized null hypersurface in a* $(n+2)$-*dimensional Lorentzian manifold* $(\overline{M},\overline{g})$ *with constant sectional curvature* *k*, $(N-\xi)$ *be a proper conformal Killing field with conformal factor* 2*λ* *and* $\lambda= \frac{1}{n+2}\operatorname{div}^{\overline{g}}(N-\xi)$. *Then for all* $X\in\mathscr{S}^{0}_{x}(1)$ *the following holds*:

94 $$ \begin{aligned}[b]\operatorname{Ric}(X) \leq{}& nk+\frac{1}{4}(n+1)^{2} \Vert H \Vert _{\overline{g}_{\eta}}^{2}-\bigl(R^{0}(N,X)X\bigr)\cdot\lambda-2 \bigl\langle A^{\star}_{\xi}X, A_{N}X\bigr\rangle \\ &-\lambda \bigl[ \langle A_{N}X,X\rangle -\bigl\langle A^{\star}_{\xi}X, X\bigr\rangle \bigr] + \bigl\{ \xi(\lambda) - \lambda^{2} +\lambda\bigl[\operatorname{tr}A_{N} -\operatorname{tr}A^{\star}_{\xi}\bigr] \bigr\} \langle X,X\rangle \\ &+\lambda\eta(X) \bigl[ \tau^{N}(X)-\lambda\eta(X) \bigr]+ \bigl[ \bigl\langle A^{\star}_{\xi}X,X\bigr\rangle -\langle A_{N}X,X\rangle+\tau^{N}(X)\eta(X) \bigr]\operatorname{tr}A^{\star}_{\xi}\\ &-\bigl\langle \bigl(\nabla_{\xi}A_{\xi}^{\star}\bigr) (X),X\bigr\rangle + 3 \bigl[ \bigl(\nabla _{X}\tau^{N}\bigr) (X) +\bigl\langle (\nabla_{\xi}A_{N}) (X),X\bigr\rangle -\bigl( \nabla_{\xi}\tau^{N}\bigr) (X)\eta (X) \bigr].\end{aligned} $$

### Corollary 5.2

*Let* $(M, g,N )$ *be a closed and conformal screen* (*with constant conformal factor* *φ*) *normalized null hypersurface in* $(n+2)$-*dimensional Lorentzian manifold* $(\overline{M},\overline{g})$, *and* $(N-\xi)$ *be a proper conformal Killing field with conformal factor* 2*λ* *and* $\lambda= \frac{1}{n+2}\operatorname{div}^{\overline{g}}(N-\xi)$. *Then*, *for all* $X\in\mathscr{S}^{0}_{x}(1)$, *the following holds*:

95 $$ \begin{aligned}[b]\operatorname{Ric}(X) \leq{}& \overline{\operatorname{Ric}}_{T_{x}M}(X)+ \frac{1}{4}(n+1)^{2} \Vert H \Vert _{\overline{g}_{\eta}}^{2}- \bigl(R^{0}(N,X)X\bigr)\cdot\lambda -2\varphi \bigl\Vert A_{\xi}^{\star}X \bigr\Vert _{\overline{g}}^{2} \\ & + \bigl\{ \xi(\lambda)-\lambda^{2} +\lambda(\varphi-1)\operatorname{tr}A^{\star}_{\xi}\bigr\} \Vert X \Vert _{\overline{g}}^{2}-\lambda^{2} \eta^{2}(X) \\ &+(3\varphi-1)\bigl\langle \bigl(\nabla_{\xi}A^{\star}_{\xi}\bigr) (X),X\bigr\rangle +\bigl[3(\xi\cdot\varphi)-\lambda(\varphi-1)+(1- \varphi)\operatorname{tr}A^{\star}_{\xi}\bigr]\bigl\langle A^{\star}_{\xi}X,X\bigr\rangle . \end{aligned}$$

*The equality case holds for all* $X\in\mathscr{S}^{0}_{x}(1)$ *if and only if either* *x* *is a totally geodesic point or* $n=1$.

### Proof

In the closed normalized and conformal screen case, it well-known in Definition 2.3 that

96 $$ A_{N}= \varphi A^{\star}_{\xi}. $$

By Lemma 2.2 in [14], the 1-form $\tau^{N}$ vanishes identically. Putting (96) in (90) and taking acount of $\tau^{N}=0$, we have

$$\begin{aligned} \operatorname{Ric}(X) \leq{}& \overline{\operatorname{Ric}}_{T_{x}M}(X)+ \frac{1}{4}(n+1)^{2} \Vert H \Vert _{\overline{g}_{\eta}}^{2}- \bigl(R^{0}(N,X)X\bigr)\cdot\lambda -2 \varphi\bigl\langle A^{\star}_{\xi}X, A^{\star}_{\xi}X\bigr\rangle \\ &-\lambda(\varphi-1)\bigl\langle A^{\star}_{\xi}X, X\bigr\rangle + \bigl\{ \xi(\lambda) -\lambda^{2} +\lambda\bigl[(\varphi- 1)\operatorname{tr}A^{\star}_{\xi}\bigr] \bigr\} \langle X,X\rangle \\ &-\lambda^{2}\eta^{2}(X)+ (1-\varphi)\bigl\langle A^{\star}_{\xi}X,X\bigr\rangle \operatorname{tr}A^{\star}_{\xi}\\ &-\bigl\langle \bigl(\nabla_{\xi}A_{\xi}^{\star}\bigr) (X),X\bigr\rangle + 3\bigl\langle \bigl(\nabla_{\xi}\varphi A^{\star}_{\xi}\bigr) (X),X\bigr\rangle .\end{aligned} $$

 □

### Remark 5.1

In the conformal sreen case with conformal factor $\varphi= 1$ we have $\tau^{N}=0$, $\operatorname{div}^{\overline{g}}N = \operatorname{div}^{\overline{g}}\xi$, $\lambda= 0$, $A_{N}^{\eta}= A_{N}=A_{\xi}^{\star}$. Hence the following holds:

### Corollary 5.3

*Let* $(M, g,N )$ *be a closed and conformal screen* (*with conformal factor* $\varphi=1$) *normalized null hypersurface in* $(n+2)$-*dimensional Lorentzian manifold* $(\overline{M},\overline{g})$, $(N-\xi)$ *be a proper conformal Killing field with conformal factor* 2*λ* *and* $\lambda= \frac{1}{n+2}\operatorname{div}^{\overline{g}}(N-\xi)$. *Then*, *for all* $X\in\mathscr{S}^{0}_{x}(1)$, *the following holds*:

97 $$ \operatorname{Ric}(X) \leq \overline{\operatorname{Ric}}_{T_{x}M}(X)+ \frac{1}{4}(n+1)^{2} \Vert H \Vert _{\overline{g}}^{2} -2\bigl( \bigl\Vert A_{\xi}^{\star}X \bigr\Vert _{\overline{g}}^{2} -\bigl\langle \bigl(\nabla_{\xi}A^{\star}_{\xi}\bigr) (X),X\bigr\rangle \bigr). $$

*The equality case holds for all* $X\in\mathscr{S}^{0}_{x}(1)$ *if and only if either* *x* *is totally geodesic point or* $n=1$.

We give now a basic inequality for shape operator and the squared mean curvature of a null hypersurface in the following.

### Theorem 5.2

*Let* $(M, g,N )$ *be a closed normalized null hypersurface in* $(n+2)$-*dimensional Lorentzian manifold* $(\overline{M},\overline{g})$, $N-\xi$ *be a proper conformal Killing field with conformal factor* 2*λ* *and* $\lambda= \frac{1}{n+2}\operatorname{div}^{\overline{g}}(N-\xi)$. *Then*, *for all* $X\in\mathscr{S}^{0}_{x}(1)$, *we have the following*.

98 $$ \begin{aligned}[b]\frac{1}{4}(n+1)^{2} \Vert H \Vert _{\overline{g}_{\eta}}^{2} \geq{}& \bigl[g(A_{N} X,X) -\tau ^{N}(X)\eta(X)\bigr]\operatorname{tr}A_{N} -g(A_{N} X, A_{N}X) \\ &- 2\lambda\tau^{N}(X)\eta(X)+\lambda^{2} \eta(X)^{2}.\end{aligned} $$

*The equality case is true for all unit vectors in* $X\in\mathscr {S}^{0}_{x}(1)$, $x\in M$, *if and only if either* *M* *is totally geodesic or* $n=1$.

### Proof

The null hypersurface equipped of Riemannian metric $g_{\eta}$ is Riemannian. Using (64) in (92), we have, for all $X\in \mathscr {S}^{0}_{x}(1)$,

$$ \overline{\operatorname{Ric}}^{\eta}_{(T_{p}M)}(X)- g_{\eta}\bigl(A^{\eta }_{N}X,A^{\eta}_{N}X \bigr)+ g_{\eta}\bigl(A^{\eta}_{N}X,X \bigr)\operatorname{tr}A^{\eta}_{N}\leq\overline{\operatorname {Ric}}^{\eta}_{(T_{p}M)}(X) + \frac{1}{4}(n+1)^{2} \Vert H \Vert _{\overline{g}_{\eta}}^{2}, $$

which gives the following inequality:

99 $$ \frac{1}{4}(n+1)^{2} \Vert H \Vert _{\overline{g}_{\eta}}^{2} \geq g_{\eta}\bigl(A_{N}^{\eta}X,X\bigr)\operatorname{tr}\bigl(A_{N}^{\eta}\bigr) -g_{\eta}\bigl(A_{N}^{\eta}X, A_{N}^{\eta}X\bigr). $$

Putting (72) of Lemma 4.1 into (99), we have (98). □

### Corollary 5.4

*Let* $(M, g,N )$ *be a closed and conformal screen* (*with conformal factor* $\varphi=1$) *normalized null hypersurface in* $(n/+2)$-*dimensional Lorentzian manifold* $(\overline{M},\overline{g})$, $(N-\xi)$ *be a proper conformal Killing field with conformal factor* 2*λ* *and* $\lambda= \frac{1}{n+2}\operatorname{div}^{\overline{g}}(N-\xi)$. *Then*, *for all* $X\in\mathscr{S}^{0}_{x}(1)$, *the following holds*:

100 $$ \frac{1}{4}(n+1)^{2} \Vert H \Vert _{\overline{g}}^{2} \geq g\bigl(A_{\xi}^{\star}X,X \bigr)\operatorname{tr}(A_{\xi}) -g\bigl(A^{\star}_{\xi}X, A_{\xi}^{\star}X\bigr). $$

Now, to establish inequality between the extrinsic scalar curvature of *M* and the scalar curvature of $(\overline{M},\overline{g})$, we shall need the following lemma.

### Lemma 5.1

[17] *If* $a_{1},\ldots, a_{n} $ *are real numbers then*

101 $$ \frac{1}{n}\Biggl( \sum_{i=1}^{n} a_{i}\Biggr)^{2}\leq\sum_{i=1}^{n} a_{i}^{2}, $$

*with equality if and only if* $a_{1}=\cdots=a_{n}$.

### Theorem 5.3

*Let* $(M^{n+1},g,N)$ *be a closed normalized null hypersurface in a Lorentzian* $(n+2)$-*manifold* $(\overline{M},\overline{g})$, $N-\xi$ *be a proper conformal Killing field*. *Then at every point* $x\in M$, *the following holds*:

102 $$ \begin{aligned}[b]r^{0}(x)\leq{}&\overline{r}(x) + \operatorname{div}^{\overline{g}_{\eta }} \tau^{N^{\sharp_{\eta}}} +\operatorname{tr}(\nabla_{\xi}A_{N}) - \operatorname{tr}\bigl(A^{\star}_{\xi}A_{N}\bigr)- \lambda^{2}+\xi(\lambda)-N(\lambda) \\ &+\frac{n}{(n+1)}(\operatorname{tr}A_{N})^{2}+\bigl[\operatorname{tr}A^{\star}_{\xi}-\operatorname{tr}A_{N} \bigr]\operatorname{tr}A^{\star}_{\xi}-\operatorname{tr}\bigl( \nabla_{\xi}A^{\star}_{\xi}\bigr)+ \operatorname{div}^{ g_{\eta}} \tau^{N{\sharp_{\eta}}},\end{aligned} $$

*where* $r^{0}(x)$ *is the extrinsic scalar curvature of* *M* *and* $\overline{r}(x)$ *is the scalar curvature of* $(\overline{M},\overline{g})$ *at* *x*. *The equality case is obtained if and only if* $x\in M $ *is a umbilical point*.

### Proof

By Lemma 5.1, we can write

103 $$ \operatorname{tr}\bigl(A^{\eta}_{N} \bigr)^{2} \geq\frac{1}{n+1}\bigl(\operatorname{tr}A^{\eta}_{N} \bigr)^{2}. $$

The equality (66) shows that

104 $$ \operatorname{tr}\bigl(A^{\eta}_{N} \bigr)^{2} = \overline{r}^{\eta}(x)-r^{\eta }(x)+ \bigl(\operatorname{tr}A^{\eta}_{N}\bigr)^{2}. $$

Putting (104) in (103), we have

105 $$ \overline{r}^{\eta}(x)-r^{\eta}(x)+ \bigl(\operatorname{tr}A^{\eta}_{N}\bigr)^{2}\geq\frac {1}{(n+1)} \bigl(\operatorname{tr}A^{\eta}_{N}\bigr)^{2}. $$

For this purpose, we use

106 $$ -r^{\eta}(x)\geq-\frac{n}{(n+1)}\bigl(\operatorname{tr}A^{\eta}_{N} \bigr)^{2}-\overline{r}^{\eta}(x). $$

By substitution of the equality (43) in (106), we have

107 $$ \begin{aligned}[b] r^{0}(x) \leq{}& \overline{r}_{\eta}(x)+ \frac{n}{(n+1)}\bigl(\operatorname{tr}A^{\eta}_{N}\bigr)^{2} + \bigl[\operatorname{tr}A^{\star}_{\xi}-\operatorname{tr}A_{N}\bigr]\operatorname{tr}A^{\star}_{\xi}\\ &-\operatorname{tr}\bigl(\nabla_{\xi}A^{\star}_{\xi}\bigr)+ \operatorname {tr}(\nabla_{\xi}A_{N})+ \operatorname{div}^{g}_{\eta}\tau^{N^{\sharp _{\eta}}}.\end{aligned} $$

Putting (81) in (107), one has the desired result. □

### Corollary 5.5

*Let* $(M^{n+1},g,N)$ *be a closed normalized and conformal screen null hypersurface in a Lorentzian* $(n+2)$-*manifold* $(\overline{M},\overline{g})$ *with conformal factor* $\varphi=1$ *and* $N-\xi$ *be a proper conformal Killing field*. *Then*, *at every point* $x\in M$, *we have*

108 $$ r^{0}(x)\leq\overline{r}(x)+\frac{n}{(n+1)} \bigl(\operatorname{tr}A^{\star}_{\xi}\bigr)^{2}-\bigl( \operatorname{tr}\bigl(A^{\star}_{\xi}\bigr)^{2}\bigr), $$

*where* $r^{0}(x)$ *is the extrinsic scalar curvature of* *M* *and* $\overline{r}(x)$ *is the scalar curvature of* $(\overline{M},\overline{g})$ *at* *x*. *The equality case is obtained if and only if* $x\in M $ *is a totally umbilical point*.

Now, we establish the inequality which gives a general optimal relationship involving the squared mean curvature of the null hypersurface and the Chen invariants.

### Theorem 5.4

*Let* $(M^{n+1},g,N)$ *be a closed null hypersurface of* $(n+2)$-*dimensional* $n>2$ *Lorentzian manifold* $(\overline{M},\overline{g})$ *with* $\tau^{N}(\xi)= 0$, $N-\xi$ *be a proper conformal Killing field with conformal factor* 2*λ*, $\lambda= \frac {1}{n+2}\operatorname{div}^{\overline{g}}(N-\xi)$. *Then*, *for each point* $x\in M$ *and for all orthonormal vectors fields* *X*, *Y* *and* $\pi=\operatorname{Span}\{X,Y\}$, *the following holds*:

109 $$ \begin{aligned}[b] \delta_{M}(x) \leqslant{}& \overline{\delta}_{\overline{M}}(x) +n(n+1) \Vert H \Vert _{\overline{g}_{\eta}}^{2} \\ &+\inf\bigl\{ B^{N}(X,Y)^{2}-B^{N}(X,X)B^{N}(Y,Y)+B^{N}(X,X)C^{N}(Y,Y) \\ &-B^{N}(X,Y)C^{N}(X,Y)-\lambda\bigl[C^{N}(X,PX)-B^{N}(X,X) \bigr]\bigr\} \\ &+ C^{N}(X,PY)C^{N}(Y,PX)-C^{N}(Y,PY)C^{N}(X,PX)+ \bigl[\operatorname{tr}A^{\star}_{\xi}-\operatorname{tr}A_{N}\bigr]\operatorname{tr}A^{\star}_{\xi}\\ &-\operatorname{tr}\bigl(\nabla_{\xi}A^{\star}_{\xi}\bigr)+\operatorname {tr}(\nabla _{\xi}A_{N})+2 \operatorname{div}\tau^{N^{\sharp}}-\operatorname {tr}\bigl(A^{\star }_{\xi}A_{N}\bigr)-\lambda^{2} +\xi(\lambda)-N(\lambda),\end{aligned} $$

*with* $\delta_{M}(x)= r^{0}(x)- \inf K(\pi)$ *and* $\overline{\delta}_{\overline{M}}(x)=\overline{r}(x)-\inf\overline{K}(\pi)$. *The equality in* (109) *holds at* $x\in M$ *if and only if there exists an orthonormal basis* $\{ e_{0},\ldots, e_{n}\}$ *of* $T_{x}M $ *such that* $\pi= \operatorname{Span}\{ e_{1},e_{2}\}$ *and the shape operator* $A_{N}^{\eta}$ *becomes of the form*

110 $$ A_{N}^{\eta} = \left ( \textstyle\begin{array}{c@{\quad}c@{\quad}c} a&0& 0 \\ 0& b& 0 \\ 0& 0& (a+b)I_{n-1} \end{array}\displaystyle \right ). $$

### Proof

By the equality (65) and (66) we have

111 $$\begin{aligned}[b] r^{\eta}(x)- K_{\eta}(\pi) ={}& \overline{r}^{\eta}(x)- \operatorname {tr}\bigl(A_{N}^{\eta}\bigr)^{2} + \bigl(\operatorname{tr}A_{N}^{\eta}\bigr)^{2} -\overline{K_{\eta}}( \pi) \\ &+g_{\eta}\bigl(A^{\eta}_{N}X,Y\bigr)g_{\eta}\bigl(A^{\eta}_{N}Y,X\bigr)- g_{\eta}\bigl(A^{\eta}_{N}Y,Y\bigr)g_{\eta}\bigl(A^{\eta}_{N}X,X\bigr).\end{aligned} $$

By Lemma 5.1, we can write

112 $$ \operatorname{tr}\bigl(A_{N}^{\eta}\bigr)^{2}\geqslant\frac{1}{(n+1)}\bigl(\operatorname{tr}A_{N}^{\eta}\bigr)^{2}. $$

In (66), we have

113 $$ \begin{aligned}[b]\operatorname{tr}\bigl(A_{N}^{\eta}\bigr)^{2} ={}& \overline{r}^{\eta}(x)- \overline {K_{\eta}}(\pi) +g_{\eta}\bigl(A^{\eta}_{N}X,Y \bigr)g_{\eta}\bigl(A^{\eta}_{N}Y,X\bigr) \\ &- g_{\eta}\bigl(A^{\eta}_{N}Y,Y\bigr)g_{\eta}\bigl(A^{\eta}_{N}X,X\bigr)+\bigl(\operatorname{tr}A_{N}^{\eta}\bigr)^{2} -r^{\eta}(x)+K_{\eta}(\pi) .\end{aligned} $$

Equation (112) with (113) gives

114 $$\begin{aligned}& \begin{aligned}[b] \frac{1}{(n+1)}\bigl(\operatorname{tr}A_{N}^{\eta}\bigr)^{2} \leq{}& \overline{r}^{\eta}(x)- \overline {K}_{\eta}(\pi) +g_{\eta}\bigl(A^{\eta}_{N}X,Y\bigr)g_{\eta}\bigl(A^{\eta}_{N}Y,X\bigr) \\ &- g_{\eta}\bigl(A^{\eta}_{N}Y,Y\bigr)g_{\eta}\bigl(A^{\eta}_{N}X,X\bigr)+\bigl(\operatorname{tr}A_{N}^{\eta}\bigr)^{2} -r^{\eta}(p)+K_{\eta}(\pi) ,\end{aligned} \end{aligned}$$

115 $$\begin{aligned}& \begin{aligned}[b]r^{\eta}(x)-K_{\eta}(\pi) \leq{}& \overline{r}^{\eta}(x)- \overline {K}_{\eta}(\pi) +g_{\eta}\bigl(A^{\eta}_{N}X,Y \bigr)g_{\eta}\bigl(A^{\eta}_{N}Y,X\bigr) \\ &- g_{\eta}\bigl(A^{\eta}_{N}Y,Y\bigr)g_{\eta}\bigl(A^{\eta}_{N}X,X\bigr)+\bigl(\operatorname{tr}A_{N}^{\eta}\bigr)^{2} - \frac{1}{(n+1)}\bigl(\operatorname{tr}A_{N}^{\eta}\bigr)^{2} \\ \leq{}& \overline{r}^{\eta}- \overline{K}_{\eta}(\pi) +g_{\eta}\bigl(A^{\eta}_{N}X,Y\bigr)g_{\eta}\bigl(A^{\eta}_{N}Y,X\bigr) \\ &- g_{\eta}\bigl(A^{\eta}_{N}Y,Y\bigr)g_{\eta}\bigl(A^{\eta}_{N}X,X\bigr)+ \frac {n}{(n+1)} \bigl(\operatorname{tr}A_{N}^{\eta}\bigr)^{2},\end{aligned} \end{aligned}$$

which gives

116 $$ \begin{aligned}[b]r^{\eta}(x) -K_{\eta}(\pi)\leqslant{}& \overline{r}^{\eta}(x)-\overline{K}_{\eta}(\pi) +n(n+1) \Vert H \Vert _{\overline{g}_{\eta}}^{2} \\ &+g_{\eta}\bigl(A^{\eta}_{N}X,Y\bigr)g_{\eta}\bigl(A^{\eta}_{N}Y,X\bigr)- g_{\eta}\bigl(A^{\eta}_{N}Y,Y\bigr)g_{\eta}\bigl(A^{\eta}_{N}X,X\bigr).\end{aligned} $$

With

$$ \bigl(\operatorname{tr}A_{N}^{\eta}\bigr)^{2} = (n+1)^{2} \Vert H \Vert _{\overline{g}_{\eta}}^{2}. $$

Putting (43), (44), (81) and (89) in (116), we have

117 $$\begin{aligned} r^{0}(x)-\inf K(\pi) \leqslant{}& \overline{r}(x)- \inf \overline{K}(\pi) +n(n+1) \Vert H \Vert _{\overline{g}_{\eta}}^{2} \\ &+\inf\bigl\{ B^{N}(X,Y)^{2}-B^{N}(X,X)B^{N}(Y,Y)+B^{N}(X,X)C^{N}(Y,Y) \\ &-B^{N}(X,Y)C^{N}(X,Y)-\lambda\bigl[C^{N}(X,PX)-B^{N}(X,X) \bigr]\bigr\} \\ &+ C^{N}(X,PY)C^{N}(Y,PX)-C^{N}(Y,PY)C^{N}(X,PX) \\&+ \bigl[\operatorname{tr}A^{\star}_{\xi}-\operatorname{tr}A_{N}\bigr]\operatorname{tr}A^{\star}_{\xi} \\ &-\operatorname{tr}\bigl(\nabla_{\xi}A^{\star}_{\xi}\bigr)+\operatorname {tr}(\nabla _{\xi}A_{N})+2 \operatorname{div}\tau^{N^{\sharp}}-\operatorname {tr}\bigl(A^{\star }_{\xi}A_{N}\bigr) \\&-\lambda^{2} +\xi(\lambda)-N(\lambda). \end{aligned}$$

 □

By (18), we have the following.

### Corollary 5.6

*Let* $(M^{n+1},g,N)$ *be a closed normalized and conformal screen null hypersurface of* $(n+2)$-*dimensional* $n>2$ *Lorentzian manifold* $(\overline{M},\overline{g})$ *with conformal factor* $\varphi=1$, $\tau^{N}(\xi)=0$ *and* $N-\xi$ *be a proper conformal Killing field*. *Then*, *for each point* $x\in M$, *for all orthonormal vectors* *X*, *Y* *and* $\pi=\operatorname{Span}\{ X,Y\}$, *the following holds*:

118 $$ \begin{aligned}[b]\delta_{M}(x) \leqslant{}& \overline{\delta}_{\overline{M}}(x) + n(n+1) \Vert H \Vert _{\overline{g}}^{2}+ \bigl\langle A_{\xi}^{\star}X,Y\bigr\rangle ^{2} \\ &- \bigl\langle A_{\xi}^{\star}X,X\bigr\rangle \bigl\langle A^{\star}_{\xi}Y,Y\bigr\rangle -\bigl(\operatorname{tr} \bigl(A^{\star}_{\xi}\bigr)^{2}\bigr)\end{aligned} $$

*with* $\delta_{M}(x)= r^{0}(x)- \inf K(\pi)$ *and* $\overline{\delta}_{\overline{M}}(x)=\overline{r}(x)- \inf\overline{K}(\pi)$. *The equality in* (118) *holds at* $x\in M$ *if and only if there exists an orthonormal basis* $\{ e_{0},\ldots, e_{n}\}$ *of* $T_{x}M $ *such that* $\pi= \operatorname{Span}\{ e_{1},e_{2}\}$ *and the shape operator* $A_{\xi}^{\star}$ *becomes of the form*

119 $$ A_{\xi}^{\star} = \left ( \textstyle\begin{array}{c@{\quad}c@{\quad}c} a&0 & 0 \\ 0& b & 0 \\ 0& 0 & (a+b)I_{n-1} \end{array}\displaystyle \right ). $$

### Remark 5.2

Since the sectional curvature of null hypersurface equipped with associated Riemannian metric is symmetric, we can denote the scalar curvature by

120 $$ \Upsilon^{\eta}(x)= \sum_{1\leq i< j\leq n+1} K_{\eta}(e_{i},e_{j}) =\frac{1}{2} r^{\eta}. $$

By (120), the equalities (43) and (66) become

$$\begin{aligned}& \Upsilon^{\eta} = r^{0}-\frac{1}{2}\bigl\{ \bigl[\operatorname{tr} A^{*}_{\xi}- \operatorname{tr} A_{N}\bigr]\operatorname{tr} A^{*}_{\xi}- \operatorname{tr}\bigl(\nabla_{\xi}A^{*}_{\xi}\bigr)+\operatorname{tr}(\nabla_{\xi}A_{N})+ \operatorname{div}^{g}\tau^{N}{^{\sharp_{\eta}}}\bigr\} , \\& \overline{r}^{\eta}(x)=\Upsilon^{\eta}(x)+\frac{1}{2}\bigl[\operatorname{tr}\bigl(\bigl(A^{\eta}_{N}\bigr)^{2}\bigr)- \bigl(\operatorname{tr}A^{\eta}_{N}\bigr)^{2}\bigr]; \end{aligned}$$

and the inequalities (108) and (118) become

121 $$\begin{aligned}& r^{0}(x)\leq\overline{r}(x)+\frac{n}{2(n+1)}\bigl(\operatorname{tr}A^{\star}_{\xi}\bigr)^{2}-\frac{1}{2}\bigl(\operatorname{tr}\bigl(A^{\star}_{\xi}\bigr)^{2}\bigr), \end{aligned}$$

122 $$\begin{aligned}& \delta_{M}(x) \leqslant\overline{\delta}_{\overline{M}}(x) + \frac{1}{2(n+1)}\|H\|_{\overline{g}}^{2}+\bigl\langle A_{\xi}^{\star}X,Y\bigr\rangle ^{2} \\& \hphantom{\delta_{M}(x) \leqslant{}}{}- \bigl\langle A_{\xi}^{\star}X,X\bigr\rangle \bigl\langle A^{\star}_{\xi}Y,Y\bigr\rangle -\bigl(\operatorname{tr}\bigl(A^{\star}_{\xi}\bigr)^{2}\bigr). \end{aligned}$$

### Application example

One important application of the Chen–Ricci inequalities for null hypersurfaces is to provide solutions to the basic problem of minimal immersions: given a null hypersurface $M^{n+1}$ in Lorentzian manifolds $\overline{\Bbb{ R}}^{n+2}_{1}$, what are the necessary conditions for *M* to admit a minimal isometric immersion in a semi-Euclidean space? As an application of our results, we have the following topological obstruction to minimal isometric immersions of null hypersurface in Lorentzian manifolds.

#### Theorem 5.5

*Let* $(M^{n+1},g,N)$ *be a closed normalized and conformal screen null hypersurface of* $(n+2)$-*dimensional* $n>2$ *Lorentzian manifold* $(\overline{M},\overline{g})$ *with conformal factor* $\varphi=1$, $N-\xi$ *be a proper conformal Killing field*. *If for all* $x\in M$ *and* $X\in\mathscr{S}(N)^{0}_{x}(1)$

123 $$ \begin{aligned}[b] \operatorname{Ric}(X,Y)\leq{}&\bigl[\bigl\langle A^{*}_{\xi}X,Y\bigr\rangle - \langle A_{N}X,Y\rangle+ \tau^{N}(X)\eta(Y) \bigr] \operatorname{tr}A^{*}_{\xi} \\ &-\bigl\langle \bigl(\nabla_{\xi}A^{*}_{\xi}\bigr) (X),Y \bigr\rangle +\bigl\langle (\nabla_{\xi}A_{N}) (X),Y \bigr\rangle \\ &-\bigl(\nabla_{\xi}\tau^{N}\bigr) (X)\eta(Y) + \bigl( \nabla_{X}\tau^{N}\bigr) (Y),\end{aligned} $$

*then the null hypersurface admits a minimal isometric immersion in Lorentzian manifold* $\Bbb{R}_{1}^{n}$ *and the null hypersurface is non*-*compact*.

#### Proof

The null hypersurface equipped with associated Riemannian metric $g_{\eta}$ is a Riemannian hypersurface. By Nash theorem, we can immerse this in a space Euclidian. By the equality (39), (123) is equivalent to say that $\operatorname{Ric}^{\eta}(X,Y)\leq0$. Given that the null hypersurface is a closed normalized and conformal screen with conformal factor $\varphi=1$, the associated Riemannian metric $g_{\eta}$ agrees with the degenerate metric *g*, the 1-form $\tau^{N}$ vanishes identically and by Definition 2.3 $A_{N}=A^{\star}_{\xi}$. This shows that $\operatorname{Ric}(X,X)\leq0$, which is a necessary condition for *M* to admit a minimal immersion in a $\Bbb{R}_{1}^{n}$.The equality hold if and only if the submanifold is totally geodesic. A well-known result of Beltrami [4] proves that the Laplacian ▵ of *M* satisfies

124 $$ \vartriangle\varphi=-(n+1)H,n+1,\quad n+1=\dim M $$

where $\varphi: M \rightarrow E^{m}$ denotes the immersion and *H* is the mean curvature vector of *M*. Since the only harmonic functions on compact Riemannian manifolds are constant functions, it follows immediately from Eq. (124) that each minimal hypersurface is non-compact. □

## Conclusion

In this paper, using rigging techniques we isometrically immerse the null hypersurface equipped with the associated Riemannian metric into a Riemannian manifold suitably constructed on the Lorentzian manifold. Through this immersion, we establish the Chen–Ricci inequalities of the null hypersurface in Lorentzian manifold. As an application, we give a topological obstruction to the minimal isometric immersion of the null hypersurface in an ambient Lorentzian.
